# NMR-based structural integrity analysis of therapeutic monoclonal antibodies: a comparative study of Humira and its biosimilars

**DOI:** 10.1080/19420862.2025.2551208

**Published:** 2025-09-08

**Authors:** Donna Baldisseri, Shen Luo, Christelle Anne F. Ancajas, Uriel Ortega-Rodriguez, Christian Fischer, Guozhang Zou, Jianghong Gu, David Keire, Martial Piotto, Baolin Zhang

**Affiliations:** a Application Laboratory, Bruker BioSpin Corp, Billerica, MA, USA; b Office of Pharmaceutical Quality Research, Office of Pharmaceutical Quality, Center for Drug Evaluation and Research, Food and Drug Administration, Silver Spring, MD, USA; c Pharma Division, Bruker BioSpin Corp, Ettlingen, Germany; d Pharma Division, Bruker BioSpin Corp, Wissembourg, France

**Keywords:** monoclonal antibody, Humira, Adalimumab, biosimilar, analytical comparability, higher order structure, NMR, photostability

## Abstract

The analytical comparability of biologic products and their biosimilars, including higher-order structure (HOS) assessment, ensures product quality and is required for regulatory approval. In this study, nuclear magnetic resonance (NMR) spectroscopy was used to evaluate the HOS of Humira (adalimumab) and its biosimilars under normal and photo-stressed conditions. Under normal conditions, 1D and 2D NMR spectra showed strong structural similarity among all products. However, photo-stressed samples exhibited distinct structural differences, including increased methionine oxidation, and localized conformational changes, most notably in the reference product. These changes correlated with findings from size-exclusion chromatography, capillary isoelectric focusing, and mass spectrometry (MS), which revealed size and charge heterogeneity, as well as site-specific methionine oxidation in the heavy chains. The differences in photostability were found to be influenced by container closure systems (CCSs) and formulations. In contrast, circular dichroism spectral analysis showed minimal variation in secondary structures among stressed and unstressed samples. These results underscore the utility of NMR as a sensitive tool for comparative structural analysis of monoclonal antibodies and their biosimilars, particularly under stress conditions, and highlight the impact of formulation and CCS on product stability.

## Introduction

Biosimilars, such as monoclonal antibodies (mAbs), are biologic products that closely resemble already approved reference products, with no clinically meaningful differences in safety or potency. As cost-effective alternatives, biosimilars expand patient access to life-saving treatments. The regulatory approval process for biosimilars involves comprehensive analytical comparability assessments, as outlined in US Food and Drug Administration (FDA) guidance documents such as *Development of Therapeutic Protein Biosimilars: Comparative Analytical Assessment and Other Quality-Related Considerations*.^1^ These assessments use orthogonal methods to evaluate critical quality attributes (CQAs), including product identity, purity, post-translational modifications (PTMs), and higher-order structural (HOS) attributes.^2^

HOS plays a crucial role in comparability assessments due to its direct impact on the safety, efficacy, and immunogenicity of therapeutic proteins.^2,3^ For therapeutic mAbs, HOS encompasses their three-dimensional (3D) conformation, including secondary structures (α-helices, β-sheets, and loops), tertiary structure (polypeptide folding into a stable conformation), and quaternary structure (assembly of heavy and light chains). Alterations in HOS, influenced by manufacturing processes, storage conditions, or formulations, can disrupt protein folding or domain arrangement (e.g., antigen-binding domain, Fc effector-binding domain), potentially affecting drug-target interactions, potency, or immunogenicity. Accurate HOS assessment ensures that biosimilars perform equivalently to reference products, maintaining similar pharmacokinetic profiles and therapeutic effects.

Methods commonly used to assess HOS include spectroscopy-based techniques such as circular dichroism (CD) spectropolarimetry, Fourier-transform infrared (FTIR) spectroscopy, and nuclear magnetic resonance (NMR) spectroscopy. CD is particularly effective for evaluating polypeptide secondary structures (e.g., α-helices, β-sheets, random coils), offering qualitative or semi-quantitative assessments based on characteristic far-UV spectral bands (190–250 nm). Near-UV CD (250–300 nm) spectra are sensitive to tertiary structural changes that affect the environments of aromatic residues and disulfide bonds. Biosimilars assessed for secondary structure similarity using CD and/or FTIR spectroscopy include published examples for mAbs^4–6^ or other therapeutic proteins, such as Eprex® (epoetin-α),^7^ Nivestim®,^8^ and Zarzio® (filgrastim).^9^

NMR is a high-resolution biophysical technique used to analyze protein structure and dynamics at the atomic level. The NMR spectrum is sensitive to conformational states of a protein in solution, making it useful for assessing various aspects of HOS, from primary to quaternary levels.^10^ However, due to NMR’s sensitivity to solution conditions, accurately determining the HOS of therapeutic mAbs in formulation buffers remains challenging. The structural complexity of mAbs and the influence of formulation components, such as excipients and pH can complicate spectral interpretation. As a result, various methodologies have been developed to improve NMR spectra comparability assessment. One such approach is the 1D-^1^H NMR technique known as Protein Fingerprint by Lineshape Enhancement (PROFILE). This method uses pulsed-field gradient stimulated echo (PFGSTE) experiments to generate highly resolved spectra of intact mAbs in formulation buffers.^11^ By exploiting differences in translational diffusion coefficients, PFGSTE greatly reduces unwanted signals from water and low molecular weight excipients at the cost of a decrease in protein signal. When combined with spectral similarity analysis,^12^ PROFILE enhances comparability assessments by enabling statistical evaluation of the mAb signals using chemometric techniques. Compared to 1D NMR, 2D NMR provides higher resolution by leveraging interactions (e.g., J-couplings) between^1^H and other nuclei, such as ^13^C or ^15^N, to generate detailed spectral maps for protein HOS characterization.^13–17^ Notably, the 2D ^1^H-^13^C methyl fingerprint NMR method utilizes methyl sidechains of a subset of six of 20 natural amino acids (i.e., Ala, Val, Met, Thr, Leu, Ile), which are often buried in hydrophobic regions and serve as sensitive probes of protein folding or HOS in comparative analyses. Furthermore, the integration of multivariate chemometric techniques with NMR spectra has significantly advanced biosimilarity assessments, incorporating both 1D- and 2D-NMR methodologies.^14–18^

In this study, we used NMR to assess the HOS of therapeutic mAbs in unstressed (as formulated) and photo-stressed conditions. Humira^Ⓡ^ (adalimumab) and its biosimilars were chosen as models due to their widespread use and a need for rigorous comparability assessments in the development of biosimilar products for the mAb. Adalimumab is a recombinant human IgG1 mAb targeting tumor necrosis factor-alpha (TNF-α) that has transformed the treatment of autoimmune diseases such as rheumatoid arthritis and Crohn’s disease. Adalimumab’s success has driven extensive biosimilar development for the product.^19–22^ As of May 2025, the FDA has approved 10 Humira^Ⓡ^ biosimilars, including Amjevita^Ⓡ^ (adalimumab-atto), Cyltezo^Ⓡ^ (adalimumab-adbm), Hyrimoz^Ⓡ^ (adalimumab-adaz), Hadlima^Ⓡ^ (adalimumab-bwwd), Abrilada^Ⓡ^ (adalimumab-afzb), Hulio^Ⓡ^ (adalimumab-fkjp), Yusimry^Ⓡ^ (adalimumab-aqvh), Idacio^Ⓡ^ (adalimumab-aacf), Yuflyma^Ⓡ^ (adalimumab-aaty), and Simlandi^Ⓡ^ (adalimumab-ryvk), with additional biosimilars in development.^23–26^ By characterizing light-induced protein modifications, we provide new insights into the structural information of these therapeutic modalities.

## Results

We conducted comparative studies on three lots of the Humira® adalimumab reference product and three lots of each biosimilar included in this study (Table 1). The two biosimilars, designated BS1 and BS2, were selected based on their commercial availability and container closure systems (CCSs), including prefilled syringes and auto-injector pens. These selection criteria enabled sampling of available products while accounting for the impact of container systems on protein stability and HOS integrity. Notably, the reference adalimumab and its biosimilars differed significantly in their CCSs, particularly in the design of their pre-filled injector pens and the estimated transparent surface area of the liquid viewing window: reference adalimumab (7.5 cm^2^) > Hadlima (~5.0 cm^2^) > Yusimry (2.0 cm^2^). The pre-filled injector pens were subjected to photostress, as described in the Materials and Methods. Photostressed and unstressed samples were analyzed in parallel using NMR, CD, and mass spectrometry (MS) for oxidation, along with assays to characterize size and charge variants.

**Table 1.** The reference adalimumab and its biosimilars tested in this study, including their formulations, container closure systems (CCSs), and the estimated transparent surface area of the liquid viewing window: the reference adalimumab (7.5 cm2) > Hadlima (~ 5.0 cm2) > Yusimry (2.0 cm2).

Product and Dosage Form	Formulation	CCS and Transparent Surface Area
HUMIRA (adalimumab) Single-dose prefilled glass syringe: 40 mg / 0.8 mL	Each 0.8 mL of HUMIRA contains: adalimumab (40 mg) citric acid monohydrate (1.04 mg) dibasic sodium phosphate dihydrate(1.22 mg) mannitol (9.6 mg) monobasic sodium phosphate dihydrate(0.69 mg) polysorbate 80 (0.8 mg) sodium chloride (4.93 mg) sodium citrate (0.24 mg) Water for Injection, USP pH 5.2	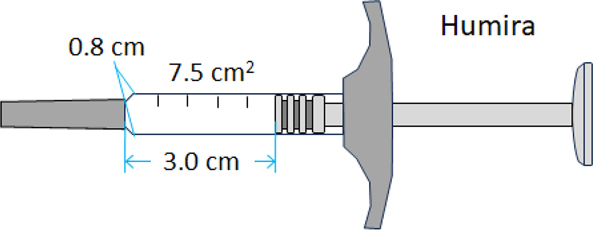
YUSIMRY (adalimumab-aqvh) Single-dose prefilled pen: 40 mg / 0.8 mL	Each 0.8 mL of YUSIMRY contains: adalimumab-aqvh (40 mg) glycine (9.61 mg) L-histidine (0.51 mg), L-histidine hydrochloride monohydrate(4.34 mg) polysorbate 80 (0.80 mg), sodium chloride (2.06 mg) Water for Injection, USP pH 5.3	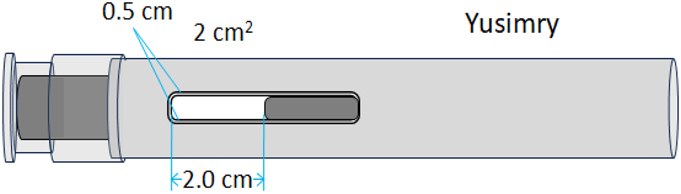
HADLIMA (adalimumab-bwwd) Single-dose prefilled glass syringe: 40 mg / 0.8 mL	Each 0.8 mL of HADLIMA contains: adalimumab-bwwd (40 mg) citric acid monohydrate (0.544 mg) L-histidine (0.96 mg) L-histidine hydrochloride monohydrate(8.64 mg) polysorbate 20 (0.64 mg) sodium citrate dihydrate (1.6 mg) sorbitol (20 mg) Water for Injection, USP pH 5.2	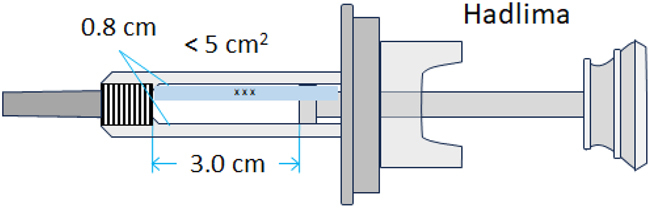

### 1D and 2D NMR spectra of adalimumab reference and biosimilars

**Figure 1.**
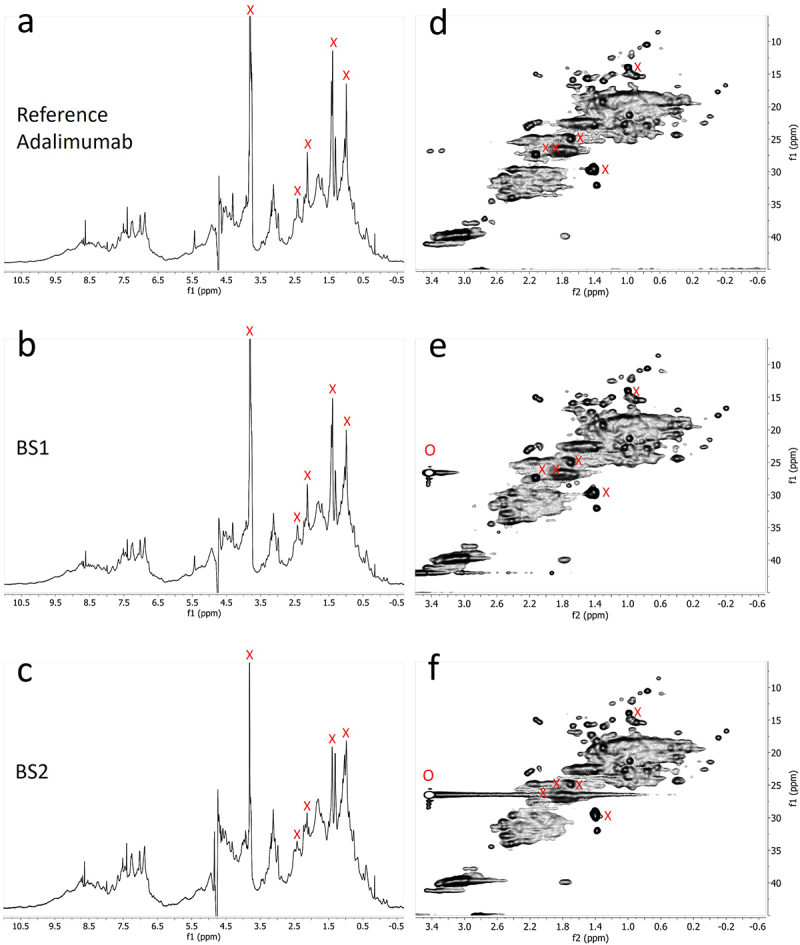
NMR spectra comparison of the reference adalimumab and biosimilars under normal conditions. 1D (a – c) and 2D (d – f) NMR spectra of the reference adalimumab, BS1, and BS2. Peaks labeled ‘X’ and ‘O’ indicate PS20/PS80 and histidine signals, respectively, which were excluded from statistical analysis.

The 1D proton (1H) diffusion-filtered NMR experiments facilitated the acquisition of spectra for the reference product and two biosimilar candidates (BS1 and BS2) in their respective formulation buffers. These spectra displayed greatly reduced signals originating from low-molecular-weight excipients, including citric acid, mannitol, and histidine, which were present in the formulations. Only residual signals from polysorbate 20 (PS20) or polysorbate 80 (PS80) excipients were observed in the 1D diffusion-filtered NMR spectra alongside those of the antibody molecules (Figure 1, *left panel*). 2D ^1^H-^13^C heteronuclear multiple quantum coherence (HMQC) NMR experiments further enhanced resolution by dispersing signals across two frequency dimensions, enabling a more detailed HOS fingerprint of the reference product and biosimilars (Figure 1, *right panel*). The 2D methyl resonance-focused technique selectively detects methyl groups from methionine, isoleucine, threonine, valine, alanine, and leucine residues in formulated mAbs by exploiting their more favorable NMR relaxation properties, leading to narrower linewidths compared to methylene or methine protons. Notably, the ^1^H-^13^C-HMQC sequence effectively attenuated signals from most excipients, with only minimal signals from histidine, PS20 or PS80 observed. The ^1^H–^13^C HMQC experiment used in our study is based on the gradient-selected XL-AlsoFast HMQC pulse sequence sequence,^27^ which enables clear detection of excipient cross-peaks in the 2D spectra. These excipient signals are easily identifiable and were excluded from subsequent analysis to focus solely on the mAb signals. To observe the methyl groups of oxidized methionine residues, the standard ^13^C spectral window of 30 ppm was expanded to 48 ppm. This broader window allowed detection of both methyl and some methylene signals from the mAb. Overall, upon visual inspection, both 1D and 2D spectra of the reference product and biosimilars appeared highly similar, further corroborating the high degree of similarity in HOS between the reference product and its biosimilars.

A quantitative analysis of NMR spectral profiles was conducted using 2D ^1^H-^13^C spectra obtained from a sample set comprising the reference product (*n* = 3), BS1 (*n* = 3), and BS2 (*n* = 3). These spectra were subjected to multivariate statistical analysis using Easy Comparability of HOS (ECHOS) and Principal Component Analysis (PCA) methodologies. The results from both chemometric approaches demonstrated minimal variations between the reference product and its biosimilar products, thereby substantiating the conclusion of high similarity in HOS among the three pharmaceutical preparations.

A Combined Chemical Shift Deviation (CCSD) analysis, based on the comparison of the position and intensity of cross-peaks in the 2D spectrum, was also performed on well-resolved cross-peaks between the unstressed reference adalimumab and the BS1 and BS2. The combined CCSD value, incorporating both ^1^H and ^13^C chemical shifts, was below 15 ppb, which is consistent with the typical range observed for biosimilars,^28^ further demonstrating the high degree of similarity between BS1, BS2, and the reference adalimumab.

### 1D and 2D NMR detection of methionine oxidation in photo-stressed samples

**Figure 2.**
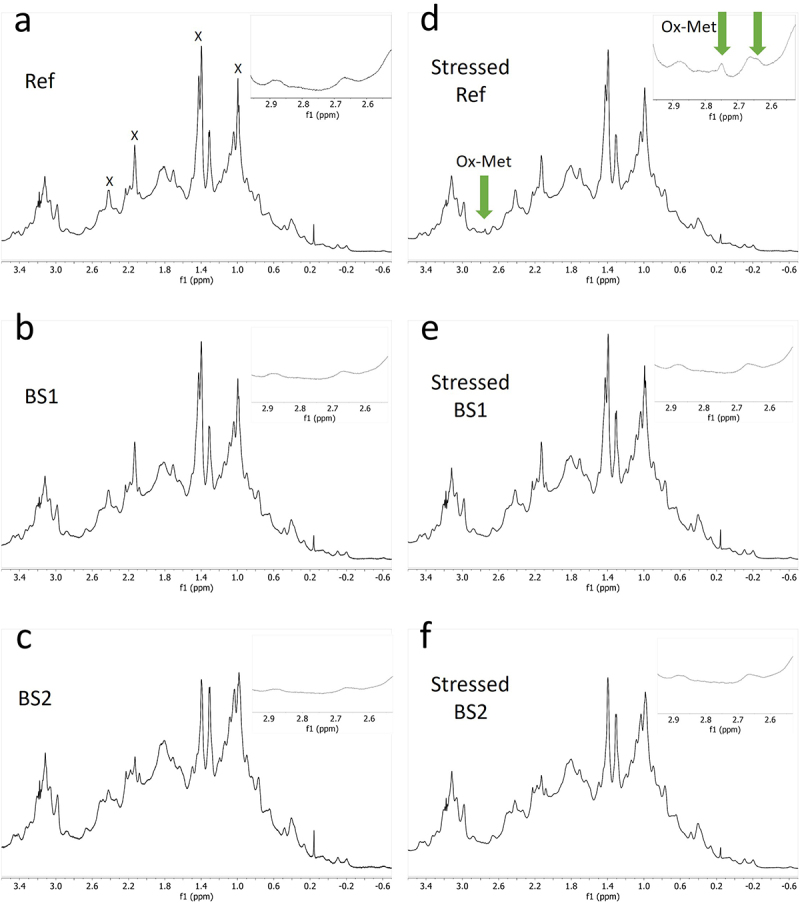
1D NMR spectra of the reference adalimumab and biosimilars under normal and photo-stressed conditions. Spectra of the reference adalimumab (a, d), BS1 (b, e), and BS2 (c, f) under normal (a – c) and photo-stressed (d – f) conditions. The peak near 2.7 ppm in panel d indicates oxidized methionine (Ox-Met). Peaks labeled ‘X’ represent PS20/PS80 signals excluded from statistical analysis.

Examination of 1D NMR spectra of photo-stressed samples revealed the presence of small additional signals around 2.7 ppm that were consistent with oxidized methionine (Ox-Met) residue chemical shifts (Figure 2).^29^ Visual inspection of the spectra showed that the amount of Ox-Met was significantly higher in the photo-stressed reference samples than in the photo-stressed BS1 and BS2 samples (Figure 2d–f).

**Figure 3.**
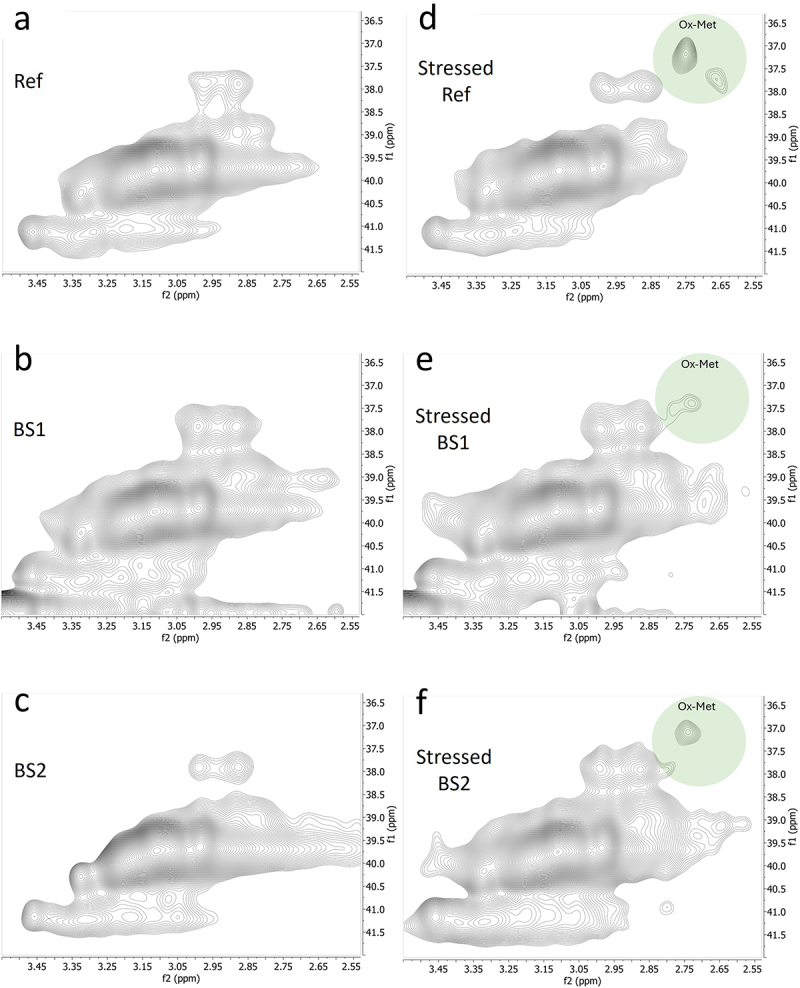
Expanded 2D NMR spectra highlighting methionine oxidation in the reference adalimumab and biosimilars. Expanded spectra of the reference adalimumab (a, d), BS1 (b, e), and BS2 (c, f) under normal (a – c) and photo-stressed (d – f) conditions, focusing on regions indicating methionine oxidation (Ox-Met).

The presence of oxidized Met was further confirmed by inspecting the 2D ^1^H-^13^C spectrum, focusing on the region where the methyl groups of Ox-Met resonate. Supplemental Figure S1 highlights the two regions of interest in the spectrum: Met and Ox-Met resonances. An expansion of the Ox-Met region of the 2D ^1^H-^13^C spectra of the reference product, BS1 and BS2, both before and after they were exposed to photo-stress, is shown in Figure 3. The additional cross peaks observed in the 2D ^1^H-^13^C spectrum of reference product indicate several sites of Met oxidation compared to BS1 and BS2. The appearance of two new signals in the (2.7, 37.5) ppm region of adalimumab’s spectrum (Figure 3d) confirmed that two Met residues underwent oxidation in the photo-stressed reference product adalimumab. In contrast, only a single Met residue was oxidized in the biosimilar comparators (BS2 and BS1), showing less oxidation of one Met residue (Figures 3e,f).

A more quantitative measurement of the relative amount of Met and Ox-Met present in each sample can be obtained by integrating the corresponding regions in the 2D ^1^H-^13^C spectrum, assuming similar relaxation properties and scalar coupling values between Met and Ox-Met methyl groups (Supplemental Figure S2). This analysis revealed that the reference product exhibited the greatest amount of methionine oxidation among the mAbs tested. Importantly, a consistent decrease in Met signal intensity was accompanied by a corresponding increase in Ox-Met intensity across the cohort, suggesting that oxidation was possibly the sole pathway affecting these observations.

### 2D NMR detection of HOS changes in photo-stressed samples

An ECHOS analysis, a pointwise comparison of two 2D ^1^H-^13^C methyl fingerprint spectra, was performed to assess structural changes. Pairwise comparisons between photo-stressed and unstressed samples of the reference adalimumab, BS1 and BS2 yielded high correlation coefficients, all above 0.97 (Supplemental Figure S3), indicating high similarity and suggesting that exposure to light had minimal impact on overall structure.

PCA of the 2D ^1^H-^13^C NMR spectra revealed little differentiation between control and photo-stressed samples, except for the photo-stressed reference adalimumab, which showed clear separation along the PC2 axis in the PC1—PC2 scores plot (Supplemental Figure S4). This separation was primarily attributed to the appearance of oxidized methionine resonances and the decrease of native Met signals. The PC1–PC3 scores plot (Supplemental Figure S5) and PCA limited to the methyl region (Supplemental Figure S6) showed similar patterns, with no additional separation. These observations further support the preservation of HOS following light exposure for BS1 and BS2.

**Figure 4.**
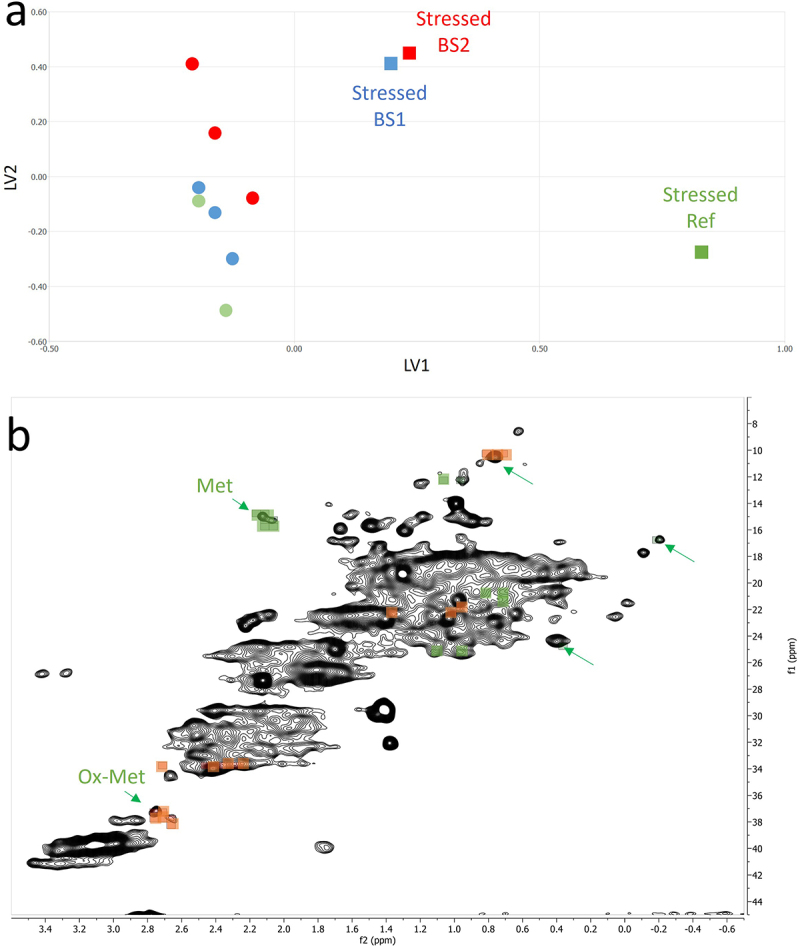
PLS analysis of 2D NMR spectra differentiating reference and photo-stressed samples. (a) PLS scores plot showing separation of unstressed (circles) and photo-stressed (squares) samples along LV1 for the reference adalimumab (green) (*n* = 3), BS1 (blue) (*n* = 4), and BS2 (red) (*n* = 4). (b) Key spectral regions driving discrimination: green squares indicate peaks enriched in reference samples; orange squares indicate peaks enriched in photo-stressed samples.

Partial Least Squares (PLS) analysis, which is more sensitive than PCA to subtle spectral differences by incorporating class information (i.e., unstressed vs. stressed), demonstrated an even clearer separation between the two groups (Figure 4a). Notably, separation along the first PLS axis (LV1) was most pronounced, with photo-stressed reference adalimumab positioned further along this axis than BS1 and BS2, consistent with earlier observations.

Analysis of the spectral regions contributing to the observed separation (referred to as “buckets”) again identified the methionine and oxidized methionine regions as major contributors (Figure 4b). However, additional spectral regions also showed significant contributions, suggesting the presence of minor HOS alterations induced by light exposure. Some regions, like the ones corresponding to the methyl resonances at (0.36, 25.85), (0.75, 10.50) and (−0.20, 16.75) ppm were consistently significant across PLS, PCA, and ECHOS analyses (Figure 4b), for distinguishing unstressed from photo-stressed samples. Notably, a subsequent PLS analysis excluding the spectral regions corresponding to Met and Ox-Met signals still achieved clear separation between control and photo-stressed samples (Supplemental Figure S7). This important finding indicates that photo-induced structural changes in mAbs extend beyond methionine oxidation, reflecting subtle but detectable modifications to the overall structure. A retrospective PCA conducted under the same conditions, excluding Met or Ox-Met signals, also revealed a separation between stressed reference adalimumab and the other samples. In our hands, the PCA and PLS analyses proved particularly useful for simultaneously analyzing the entire sample cohort. The ECHOS method, while also valuable, lacks this capability due to its pairwise comparison approach.

### 2D NMR identification of amino acid residues in adalimumab

**Figure 5.**
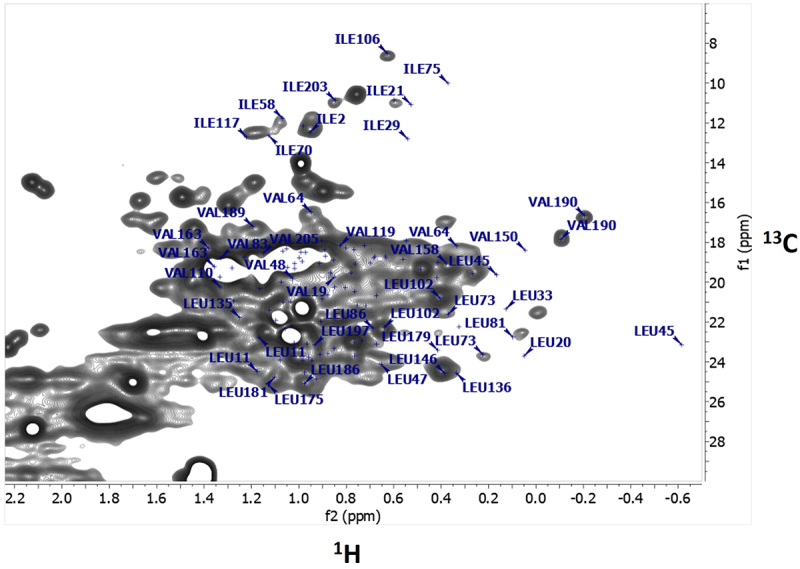
Methyl group assignments of ILV residues in the 2D ^1^H-^13^C spectrum of adalimumab. Assignments of isoleucine (I), leucine (L), and valine (V) methyl groups based on Sarkar and Aubin.^31^ the two methyl groups of VAL190 appear as distinct cross-peaks.

Sequence-specific assignments for the ILV methyl groups of the reference adalimumab antigen-binding fragment (Fab), obtained using 900 MHz NMR, were recently reported (BMRB-52274).^30,31^ In the current work, the BMRB-52274 assignments were transferred to the 2D ^1^H-^13^C spectra of intact adalimumab and are illustrated in Figure 5. These assignments were then used to identify residues showing differences in the 2D ^1^H-^13^C HMQC spectra of unstressed and photo-stressed samples. Because the current study used intact formulated mAbs measured at 600 MHz, the spectra were less resolved and showed broader peaks compared to the 900 MHz Fab data. As a result, only well-resolved peaks could be reliably used to detect residue-level HOS differences, with nine of 117 residues meeting this criterion.

Using these assignments, several peaks, previously identified as significant in differentiating between unstressed and photo-stressed samples through PCA, PLS, and ECHOS analyses, were assigned to L175/L181, L186/L78, L136/L146, I2/I51 and V190 (Supplemental Figure S3 ECHOS heat map). The syntax L175/L181 indicates that there is an ambiguity in the assignment between L175 and L181. Several additional spectral regions corresponding to assigned peaks were also flagged as important in the statistical analyses, but, because of peak overlap, their specific identities remain ambiguous. These residues are potentially indicative of local changes in the HOS of the mAb.

Since the crystal structure of adalimumab bound to its antigen TNFα (PDB entry 3WD5) has been reported,^32,33^ it is possible to identify the position of the assigned residues on the structure of the mAb and potentially obtain some information on the regions of the mAb that experience structural changes.

### Far-UV CD spectra of reference adalimumab and its biosimilars

**Figure 6.**
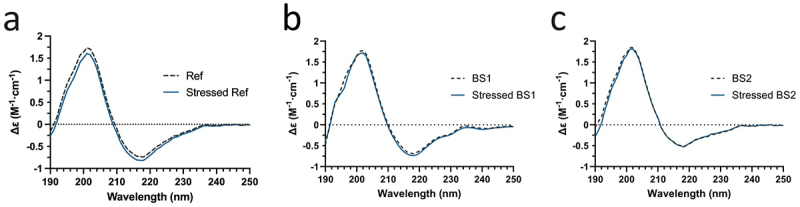
Circular dichroism analysis of the reference adalimumab and biosimilars. (a – c) Representative far-UV CD spectra of the reference adalimumab and biosimilars compared to photo-stressed samples, showing spectral similarity. Molar ellipticity is expressed as Δε (delta epsilon); M denotes molarity (see materials and methods).

The recorded Far-UV CD spectral profiles were consistent with the typical folded IgG1 mAb structure, displaying characteristic peak wavelengths with a minimum at ~217 nm and maximum at 201–202 nm. The reference adalimumab, BS1, and BS2 (dashed line) visually exhibited highly comparable CD spectral profiles, demonstrating similar characteristic features (Figure 6). When compared to their photo-stressed counterparts (blue solid line), these profiles also showed nearly complete overlap. The BeStSel program^34^ was used to quantify secondary structure content from CD molar ellipticity data and revealed similar levels of the protein’s α-helices, β-sheets, turns, and others(Table 2).The reference adalimumab, BS1, and BS2 contained comparable anti-parallel β-sheet content at 49.6, 48.4, and 50.2%, respectively. Photo-stress resulted in only a minor increase in β-sheet content (0.3%) for the reference adalimumab and slight decreases for photo-stressed BS1 (0.3%) and BS2 (1.0%). Overall, secondary structure content was similar between unstressed reference product, BS1, BS2, and their stressed counterparts, confirming that photo-stress conditions, and consequently methionine oxidation, did not alter the overall secondary structures of the reference product or its biosimilars. These results are consistent with previous reports that methionine oxidation minimally perturbs secondary structure.^35^

**Table 2.** Calculated percentages of secondary structure, showing the mean and standard deviation from four replicate spectral scans (n=4).

Secondary Structure	Reference Adalimumab (Ref)	Stressed Ref	BS1	Stressed BS1	BS 2	Stressed BS2
	Mean ± SD	Mean ± SD	Mean ± SD	Mean ± SD	Mean ± SD	Mean ± SD
α-helix	0.4 ± 0.22	0.4 ± 0.30	0.2 ± 0.10	0.2 ± 0.10	0.1 ± 0.06	0.1 ± 0.10
β-sheets (anti-parallel)	49.6 ± 1.28	49.9 ± 0.84	48.4 ± 1.70	48.1 ± 2.00	50.2 ± 1.08	49.2 ± 1.15
Turn	12.9 ± 0.44	12.5 ± 0.28	13.9 ± 0.54	13.1 ± 0.69	13.6 ± 0.21	13.9 ± 0.63
Others	37.1 ± 1.02	37.2 ± 0.48	37.7 ± 1.26	38.8 ± 1.78	36.3 ± 1.20	36.9 ± 0.86

### Size variants by SEC and CE-SDS

**Figure 7.**
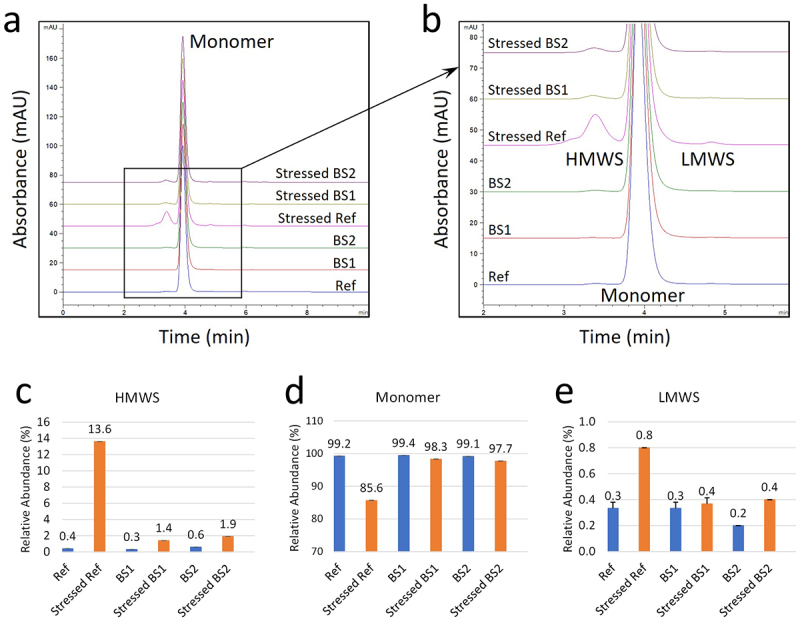
Size variant analysis of the reference adalimumab and biosimilars by SEC. (a) SEC profiles showing separation of monomer, high molecular weight species (HMWS), and low molecular weight species (LMWS) in unstressed and photo-stressed samples; boxed region enlarged in (b). (c – e) relative abundances of HMWS, monomer, and LMWS, respectively, calculated from peak areas. Error bars represent standard deviation (*n* = 3).

SEC analysis showed that adalimumab reference product and its biosimilars had similar levels of high molecular weight species (HMWS), monomers, and low molecular weight species (LMWS) (Figure 7). After photo-stress, HMWS in reference adalimumab increased from 0.4% to 13.6%, accompanied by a decrease in monomer content. In contrast, the photo-stressed biosimilars exhibited only minor increases in HMWS, ranging from 0.3% to 1.9%. LMWS increased slightly in all samples, ranging from 0.3% to 0.8%.

**Figure 8.**
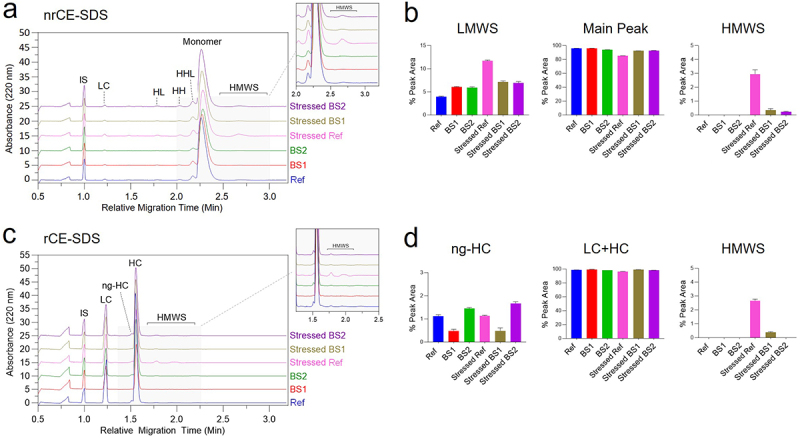
Size variant analysis by reducing (r) and non-reducing (nr) Turbo CE-SDS.

Capillary electrophoresis sodium dodecyl sulfate (CE-SDS), in both reducing and non-reducing (rCE-SDS and nrCE-SDS, respectively) modes, was performed as an orthogonal approach to assess size variants. nrCE-SDS analysis of the reference adalimumab and its biosimilars consistently showed a monomer peak with a relative migration time (RMT) of 2.3 minutes (Figure 8a). Additionally, various LMWS were detected, including light chain fragments (LC, 1.23 RMT), light-heavy fragments (HL, 1.78 RMT), light-light-heavy fragments (LLH, 2.01 RMT), and heavy-heavy-light fragments (HHL, 2.18 RMT). In stressed samples, a distinct HMWS peak appeared at 2.65 RMT, which was most prominent in stressed reference adalimumab. Quantification of size variants by nrCE-SDS showed that before photo-stress, the relative abundances of the main peak (monomer) and HMWS were similar (Figure 8b, middle and *right panel*s). The reference adalimumab contained approximately 4% LMWS, compared to 6% in BS1 and BS2 (Figure 8b, *left panel*). After photo-stress, HMWS in the reference adalimumab increased to 3%, whereas only minor increases were observed in BS1 (0.34%) and BS2 (0.22%). Photo-stress also led to an increase in LMWS from 4% to 11.7% in the reference adalimumab, while BS1 and BS2 showed only slight increases (~1%). Although SEC detected a higher HMWS level in photo-stressed reference adalimumab (13.6%), the presence of sodium dodecyl sulfate (SDS) in the CE-SDS running buffer likely disrupted most aggregates. Additionally, the high resolution of capillary electrophoresis may have enhanced the separation of LMWS, which could explain the increased LMWS detection in stressed reference adalimumab compared to SEC.

(a) nrCE-SDS electropherograms and zoomed-in view of high molecular weight species (mean of three injections). (b) Summary of nrCE-SDS results: LMWS, monomer, and HMWS. (c) rCE-SDS electropherograms showing LC, HC, and ng-HC after reduction. (d) Summary of rCE-SDS results: ng-HC, total purity (LC + HC), and HMWS.

Reducing CE-SDS revealed two major peaks corresponding to free LC (1.23 RMT) and HC (1.55 RMT), along with a minor peak at 1.51 RMT representing non-glycosylated heavy chain (ng-HC) (Figure 8c). Additionally, HMWS that persisted after reduction were detected at 1.75 RMT and 1.85 RMT (Figure 8c, zoomed insert). The purity of the reference adalimumab and its biosimilars was determined by summing the peak areas of LC and HC (Figure 8d, middle insert), revealing only minor differences between the originator and biosimilars. In stressed reference adalimumab, the purity decreased by ~3%, accompanied by a ~3% increase in HMWS, consistent with the HMWS levels observed in nrCE-SDS (Figure 8b, *right panel*). Aside from the ng-HC peak, no other LMWS were detected, suggesting that many LMWS observed in nrCE-SDS correspond to mispaired antibody forms (HL, LLH, HHL). These LMWS reduce to ng-HC (1.51 RMT), HC (1.55 RMT), and LC (1.23 RMT) upon disulfide bond reduction. Regarding ng-HC comparability, minor differences were observed among unstressed mAbs, with ng-HC levels below 1.5% in each sample (reference adalimumab: 1.12%; BS1: 0.48%; BS2: 1.5%). After photo-stress, ng-HC levels remained relatively unchanged (photo-stressed reference adalimumab: 1.13%; BS1: 0.49%; BS2: 1.7%). Taken together, these findings further support that BS1 and BS2 were less susceptible to photo-stress-induced degradation than the reference adalimumab.

### LC-MS detection of methionine oxidation

**Figure 9.**
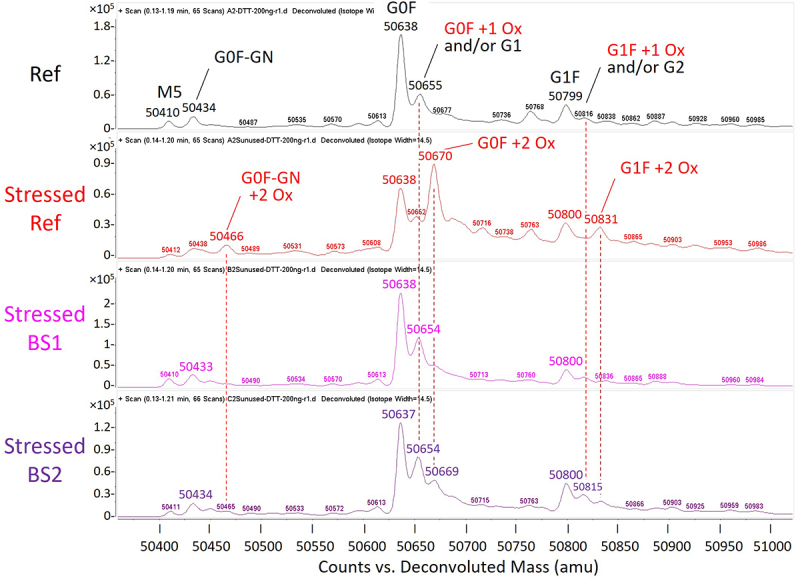
Oxidation analysis of the reference adalimumab and biosimilars by reduced LC-MS. Deconvoluted mass spectra of the heavy chain for the reference adalimumab and photo-stressed samples, analyzed by direct infusion mass spectrometry. Spectra for both light and heavy chains are provided in Supplemental Figure S8. Major peaks are labeled with attached N-glycans, and peaks with a 16 or 32 Da increase are labeled as +1 or +2 oxygen (Ox), indicating oxidation of one or two methionine residues. Note that G1 and G2 are also 16 Da larger than G0F and G1F, respectively. GN represents N-acetylglucosamine (GlcNac).

LC-MS analysis under reduced conditions identified the following N-glycans on the HC of the reference adalimumab: G0F, G1F, G0F-GN, G1, G2, and M5 (Figure 9). Photo-stress had no impact on the light chain (Supplemental Figure S8), but caused peak shifts of +32 Da (+2 oxygen) on the HC for G0F, G1F, and G0F-GN, indicating oxidation of two methionine residues. In contrast, stressed BS1 and BS2 exhibited mainly a +16 Da (+1 oxygen) shift for G0F and G1F, suggesting oxidation of a single methionine residue. This finding aligned with SEC data, suggesting that differences in oxidation susceptibility may be due to the transparent area in the CCS. Notably, BS1 demonstrated the highest photostability, correlating well with its smallest transparent surface area of 2 cm^2^ in the autoinjector pen’s viewing window, as compared to 7.5 cm^2^ in the reference adalimumab syringe, and < 5 cm^2^ in BS2 syringe that contains a painted area for texts blocking more than one-third of its transparent surface (see CCS images in Table 1).

**Figure 10.**
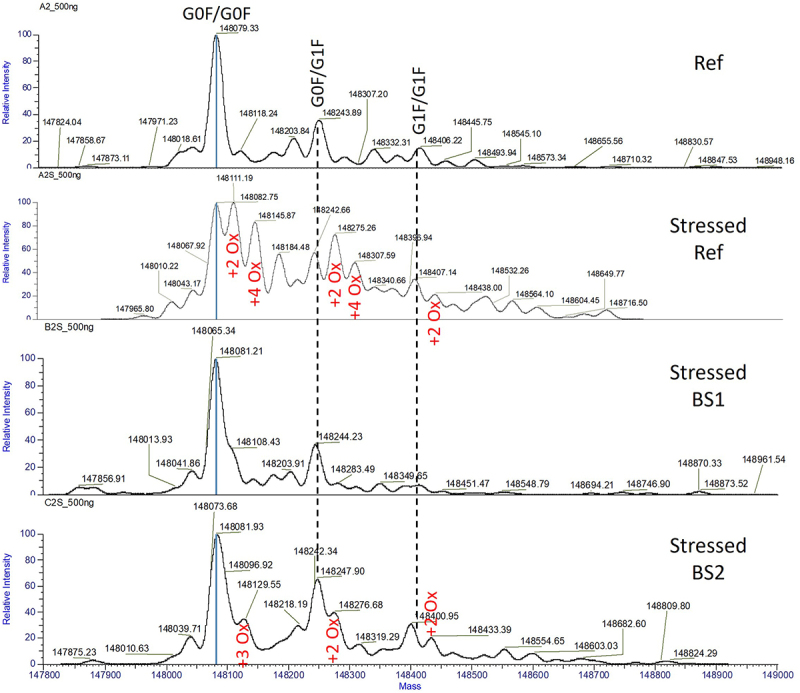
Oxidation analysis of the reference adalimumab and biosimilars by intact LC-MS. Deconvoluted mass spectra of unstressed the reference adalimumab and photo-stressed reference adalimumab, BS1, and BS2. Mirror spectra of unstressed and stressed samples are provided in Supplemental Figure S9.

Intact LC-MS identified three major glycoforms in the reference adalimumab: G0F/G0F, G0F/G1F, and G1F/G1F (Figure 10). Photo-stress induced two oxidized species, +32 Da (+2 oxygen) and +64 Da (+4 oxygen). In comparison, the two biosimilars were less susceptible to oxidation under the same stress conditions, with BS1 exhibiting the highest resistance (Figure 10 and Supplemental Figure S9).

### Charge variants by icIEF

**Figure 11.**
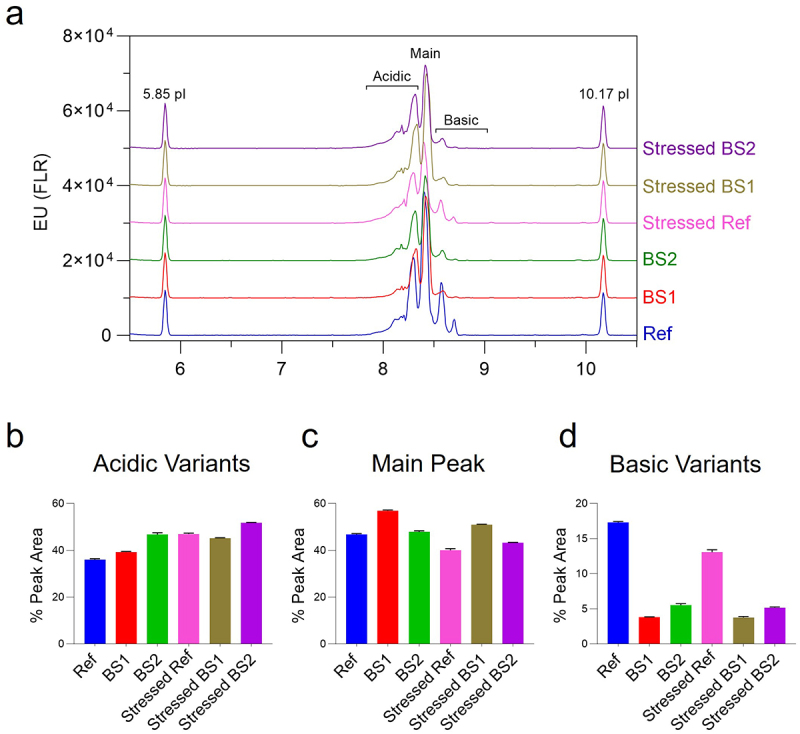
Charge variant analysis of the reference adalimumab and biosimilars by icIEF. (a) Electropherograms of unstressed and photo-stressed reference adalimumab and biosimilars, showing minor charge heterogeneity differences. Plots represent the mean of three injections. (b – d) relative abundance of acidic, main, and basic charge variants. Basic variants in the reference adalimumab were most affected by photo-stress. Bar graphs show mean ± SD (*n* = 3).

icIEF analysis identified a main charge variant with an isoelectric point (pI) of 8.4, along with a second dominant peak at pI 8.3 in all reference adalimumab samples and its biosimilars (Figure 11). Differences between unstressed reference adalimumab, BS1, and BS2 were observed in the abundance of basic variants, which resolved at pI 8.5 and 8.7. In general, photo-stress caused a slight decrease in the main charge variant (reference adalimumab: −6.75%; BS1: −5.94%; BS2: −4.66%), accompanied by a minor increase in acidic species (reference adalimumab: +10.99%; BS1: +5.97%; BS2: +5.00%). Regarding the photostability of basic variants, BS1 and BS2 showed minimal losses after photo-stress (BS1: −0.03%; BS2: −0.35%). In contrast, the reference adalimumab exhibited a more significant loss of 4.24%, likely due to degradation into acidic species. Taken together, these data suggest that BS1 and BS2 exhibited greater photostability than the reference adalimumab. Furthermore, the findings indicated that the basic charge variants in the adalimumab reference product may contribute to its lower photostability, as demonstrated by their susceptibility to degradation.

## Discussion

**Figure 12.**
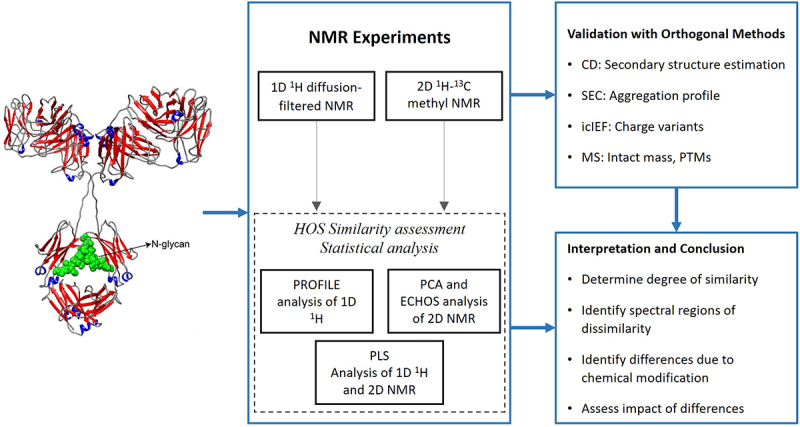
Schematic workflow of NMR and complementary analytical methods. Workflow outlining the NMR experiments, NMR data analysis, and complementary analytical methods used to assess changes in mAb structure, HOS, size variants, and charge variants. The antibody structure figure was adapted from Japelj et al.^36^ and recreated using PowerPoint.

Analytical comparability between biologics and their biosimilars is a cornerstone of regulatory evaluation, providing assurance that any observed differences between the reference originator product and the biosimilar candidate do not meaningfully impact the product’s safety or efficacy. This study focused on the HOS aspect of comparability analysis, with a particular emphasis on the use of NMR spectroscopy. Under normal, unstressed conditions, the reference adalimumab and its biosimilars demonstrated structural similarity, as evidenced by visually overlapping 1D and 2D NMR spectra. These results confirmed that the biosimilars maintained structural organization comparable to the reference product. The study also highlighted the value of orthogonal analytical methods in evaluating structural comparability. Collectively, the analytical techniques applied in this work illustrate an example of a practical workflow for HOS characterization and biosimilarity assessment (Figure 12).

The ability of analytical techniques to detect differences in HOS is a critical consideration in comparability assessment. In this work, photo-stressed adalimumab was used to assess the sensitivity of the methods to protein modifications and the resulting structural changes. Notable alterations were observed, particularly in the reference adalimumab samples compared to the available biosimilars, indicating differences in susceptibility to photo degradation. Although only a limited number of samples were tested, these differences may be attributed to variations in product formulations and CCSs. For example, while both the reference adalimumab and its biosimilars share a similar pH range (5.2–5.3), the biosimilar formulations included histidine, a buffering agent known to protect proteins from oxidation and aggregation.^37,38^ This formulation difference likely contributed to the enhanced photostability observed in the biosimilars, underscoring the importance of formulation-specific considerations in biosimilar development.

CCS design also appeared to influence product photostability. For example, Yusimry (adalimumab-aqvh) in prefilled pen exhibited superior photostability, likely due to its CCS design, which featured a smaller transparent area with a limited viewing window that reduced light exposure (Table 1). The design may mitigate photodegradation pathways, including methionine oxidation, protein aggregation, and charge variant formation (Figures 7–11). In contrast, more transparent or translucent CCS designs may allow greater light penetration, potentially accelerating degradation under the stressed conditions. Our study offers preliminary insights into photostability differences between the reference product and biosimilars, but it is limited to evaluations within CCSs. Consequently, it does not fully characterize intrinsic photostability or reflect conditions encountered during clinical use. According to ICH Q1B guidelines, a comprehensive assessment should include testing of reconstituted solutions and a broader sampling of lots to capture potential variability. In the absence of these data, the observed differences may primarily reflect packaging effects rather than formulation stability.

NMR analysis revealed chemical modifications and localized conformational changes, underscoring its sensitivity in detecting structural differences that may be overlooked by techniques such as CD spectropolarimetry. While CD showed minimal differences among samples (Figure 6), NMR provided high-resolution insights into structural perturbations (Figures 1–5), making it especially valuable for in-depth, atomic-level characterization under stress conditions. These findings were consistent with SEC and cIEF results, which indicated size and charge heterogeneity, further supporting degradation-related changes (Figures 7 and 11).

A key observation was the correlation between altered NMR spectra and methionine oxidation in the HC, as confirmed by MS (Figure 9), highlighting the impact of oxidative modifications on mAb stability. These results emphasize the importance of photostability testing in comparability assessments. Photo-induced oxidation in mAbs, particularly at methionine residues such as Met252 and Met428,^39^ can impair Fc receptor binding and effector function. Unlike chemical oxidants (e.g., hydrogen peroxide, peracetic acid, 2,2’-azobis(2-amidinopropane) dihydrochloride),^22,40,41^ which often produce nonspecific and non-physiological modifications, photo-stress more closely mimics real-world degradation pathways. NMR-based HOS analysis was instrumental in detecting site-specific conformational changes under these conditions, directly linking methionine oxidation to structural alterations. Further studies are underway to evaluate other stress factors, such as elevated temperature, which may help eliminate CCS-related variability in degradation profiles. Focus will be on determining whether methionine oxidation or other modifications impact the bioactivity of adalimumab.

Accurate HOS evaluation also necessities analyzing therapeutic proteins in their native formulation buffers rather than in simplified buffers. While a uniform buffer may simplify comparisons, uniform buffering can obscure interactions between proteins and excipients designed to stabilize them in formulation, potentially leading to misleading conclusions. By assessing the reference adalimumab and its biosimilars in their respective formulation buffers, this study offers a more accurate, real-time evaluation of structural comparability. The enhanced photostability of biosimilars, partly due to histidine, further supports the need for formulation-specific evaluations in biosimilar development.

Although the sensitivity of NMR allows the detection of differences in HOS, it is considered technically demanding and requires specialized instrumentation and expertise. Nevertheless, the atomic-level insights it provides can be highly valuable, particularly in addressing knowledge gaps related to the relationship between structure and function. Integrating NMR-based HOS analysis with orthogonal biophysical and biochemical methods, along with mechanism-of-action reflective bioassays, enables a more comprehensive assessment of how post-translational modifications (e.g., methionine oxidation) affect structural integrity and biological activity. This combined approach enhances product understanding, supports regulatory expectations, informs control strategies, and ultimately contributes to product quality and consistency of biologics and biosimilars throughout their lifecycle.

In conclusion, this study demonstrates the power and sensitivity of NMR in assessing the structural integrity of formulated mAbs and their biosimilars, especially under stress conditions. Our findings provide valuable insights into the impact of post-translational modifications, particularly methionine oxidation, on protein stability and reinforce NMR’s potential utility in biosimilar evaluations.

## Materials and methods

### Pharmaceutical antibodies

The reference product, Humira® (adalimumab), and its two biosimilars, Yusimry® (adalimumab-aqvh) and Hadlima® (adalimumab-bwwd), were obtained from commercial sources through a pharmaceutical procurement contract service. For simplicity, the biosimilars are referred as BS1 (Yusimry®) and BS2 (Hadlima®). Detailed product information including formulations and CCS formats, as listed in the respective drug labels,^42^ are provided in Table 1.

### Photo-stress

One lot of each product in its original CCS (see Table 1) underwent photostability testing per ICH Q1B guidelines Option 2.^43^ Prefilled CCSs were placed in a photostability chamber (Caron model 7545–11–2) with a condensate recirculator (Caron model CRSY-102–1) to maintain 25 ± 2°C and 65 ± 5% relative humidity. Samples were exposed to 60 hours of visible light (20 klux) and 12 hours of UV light (20 W/m^2^), meeting the ICH Q1B minimum exposure requirements of 1.2 million lux-hours of visible light and 200 W/m^2^ of UV light). The photo-stressed samples were stored at 4°C until analysis.

### Nuclear magnetic resonance spectroscopy

Samples of reference adalimumab and its biosimilars in their original formulations were analyzed by 1D ^1^H and 2D ^1^H-^13^C NMR spectroscopy. All spectra were acquired on an Avance Neo 600 MHz NMR spectrometer equipped with a 5 mm TCI (H-F/C/N) Cryoprobe (Bruker BioSpin) at 318 K (45°C), a temperature commonly used for mAbs to improve spectral resolution by narrowing signal linewidths. The NMR tubes used for the measurements were prepared by adding 0.3 mL of a formulated 50 mg/mL mAb solution and 0.015 mL of D_2_O to a 4 mm Match NMR tube (Bruker BioSpin).

1D ^1^H NMR spectra were acquired using a diffusion filter experiment based on a PFGSTE (Bruker library parameter set HOS_1DSTE and pulse sequence hos_stebpgp1s1d). This sequence is designed to strongly attenuate the signals from low molecular weight compounds present in the formulation buffer, allowing protein signals to remain in the spectrum. The water signal was also further suppressed by the PFGSTE diffusion filter. The acquisition parameters were as follows: 512 scans, 16 dummy scans, 64K time domain points, a spectral width of 21.0 ppm, pulse width of 11 μs, acquisition time of 2.6 s, relaxation delay of 4.0 s, diffusion time 100 ms, gradient strength 65 G/cm, gradient duration 3 ms (2 ×1.5 ms). The total experiment time was 1 hour.

2D ^1^H-^13^C NMR spectra were acquired using the XL-AlsoFast Heteronuclear Multiple Quantum Coherence (Bruker parameter set HOS_XLAFHMQC and pulse sequence hos_afhmqcetgpph).^27,44^ Spectra were recorded with 512 scans per free induction decay, 128 dummy scans, 818 × 180 time-domain points, a spectral width of 14 ppm in ^1^H and 48 ppm in ^13^C, a pulse width of 11 μs and a relaxation delay of 0.5 s. The total experiment time was approximately 7 hours. To assess any potential impact of the NMR conditions, we performed SEC analysis on all three unstressed products before and after NMR measurements at 45°C for 7 hours. The results showed only minor increases in HMWS (+0.1%) and LMWS (+0.2–0.3%), indicating minimal effects on the mAbs (Supplemental Figure S10).

The raw NMR data were first Fourier transformed and phase corrected using Topspin version 4.4.0 software (Bruker BioSpin). The 1D^1^H NMR spectra were processed with a 0.3 Hz exponential line broadening function and zero filled to 64K real points. The 2D ^1^H-^13^C NMR spectra were processed using an apodization function in F2 calculated by the library proc_dd.py Python program, which then applies a cosine squared function in F1. Both 1D and 2D NMR spectra were carefully referenced to the signal of the methyl group of I106, which resonates at 0.6259 and 8.6070 ppm in the 1 H and 13C dimension, respectively, to ensure reliable comparison between the different samples.^37,38^

Multivariate data analyses, including PROFILE, ECHOS, CCSD,^45^ PCA, and PLS were performed using the BioHOS plug-in of MestreNova version 15.1.0 (Mestrelab Research), on both 1D and 2D NMR spectra. Spectral regions corresponding to excipient signals and noise were excluded from the statistical analysis.

### Circular dichroism

Far-UV CD spectra of reference adalimumab and its biosimilars were measured at a protein concentration of 0.25–0.27 mg/mL in sterile water using quartz cuvettes with a 0.1 cm path length. The measurements were performed with a JASCO-1700 CD spectrophotometer at 20°C, covering a wavelength range of 190–250 nm. All samples were analyzed under the same settings, with continuous scanning at 0.1 nm intervals, a response time of 50 nm/min, an average of 3 scans, and a bandwidth of 1.0 nm. Water-only blanks were measured and subtracted from the corresponding sample readings (4 replicates). CD ellipticity values were normalized to protein concentration, path length, and the number of residues (1330). The ellipticity values between 200–250 nm were converted to Δε (M^−1^ cm^−1^) and analyzed using the BeStSel program, then plotted with GraphPad Prism 10.0.0. The secondary structure content (α-helices, β-sheets, turns, and others) was estimated by deconvoluting the far-UV CD molar ellipticity data using the BeStSel algorithm.^34^

### Size variants analysis via size exclusion chromatography

Protein size variants were quantified under native conditions using an Agilent 1290 Infinity II UHPLC system with an AdvanceBio SEC 300 Å, 2.7 μm, 4.6 × 150 mm column at 25°C. The mobile phase (200 mM potassium phosphate, 250 mM potassium chloride, pH 6.2) flowed at 0.30 mL/min. Samples (2 μL, 50 mg/mL) were injected in triplicate, followed by a mobile phase control. Runs lasted 10 minutes with detection at 280 nm. Data were analyzed using Agilent OpenLab CDS ChemStation (version C.01.10).

### Liquid chromatography-mass spectrometry

The intact mass of each sample was analyzed using a Vanquish LC system coupled to a Fusion LUMOS mass spectrometer (Thermo Fisher Scientific). Samples (1 μL of 0.5 mg/mL) were separated on a MAbPac RP column (4 μm, 2.1 × 50 mm) using mobile phases of 0.1% formic acid in water (A) and acetonitrile (B). A linear gradient from 5% to 95% B over 11.5 minutes was applied at 60°C and 0.1 mL/min. Liquid chromatography was interfaced with the mass spectrometer via a heated electrospray ionization source with a 3500 V positive voltage, sheath gas at 30 L/min, auxiliary gas at 10 L/min, and temperatures of 275°C for the ion transfer tube and vaporizer. The mass spectrometer operated in intact protein mode, with an Orbitrap resolution of 30,000, scan range m/z 500–4500, AGC target 4 × 10^5^, maximum injection time 100 ms, and source fragmentation voltage of 60 V. Data analysis was performed using BioPharma Finder 5.2 (Thermo Fisher Scientific) with time-resolved deconvolution to determine intact mass and relative abundance. Spectra were integrated using the Sliding Window algorithm (0.32 min spectrum width, 30 ppm merge tolerance) and deconvoluted using the ReSpect algorithm (charge states 10–100, 6–10 adjacent charges).

Reduced samples were analyzed using an Agilent 1260 HPLC 6520 Q-TOF. Samples (1.0 mg/mL in 50 mM ammonium bicarbonate, pH 7.8) were reduced with 50 mM dithiothreitol for 30 minutes at 37°C, diluted to 0.1 mg/mL in 0.1% formic acid, and kept at 5°C in autosampler. Direct infusion of 2 μL sample was performed using mobile phases of 0.1% formic acid, 16% isopropanol, and 2% acetonitrile. Mass correction used an internal reference ion (922.0098 Da). The Q-TOF settings were VCap 5,500 V, fragmentor 380 V, skimmer 65 V, drying gas flow 10 L/min at 350°C. Data analysis software was Agilent MassHunter (version B.05.00) with Bioconfirm add-on for mass spectra deconvolution.

### Charge variant analysis via imaged capillary isoelectric focusing

The charge heterogeneity of photo-stressed reference adalimumab and its two biosimilars was assessed using a ProteinSimple Maurice capillary electrophoresis instrument (ProteinSimple, San Jose, CA). Briefly, 35 µg of each protein was diluted in 200 µL of an ampholyte mixture containing 0.35% methylcellulose (Cat# 101876, Bio-Techne), 3% pH 8–10.5 Pharmalyte (Cat# 17045501, Cytiva, Washington, D.C.), 1% pH 5–8 Pharmalyte (Cat# 17045301, Cytiva), 3.2 M urea (Cat# 51457, Sigma-Aldrich), 5 mM L-arginine (Cat# 042–691, Bio-Techne), 10 mM iminodiacetic acid (Cat# 220000, Sigma-Aldrich), 1% pI marker 5.85 (Cat# 046–030, Bio-Techne), and 1% pI marker 10.17 (Cat# 046–035, Bio-Techne).

Charge variant separation was performed using a Maurice cIEF cartridge (Cat# PS-MC02-C, ProteinSimple) under the following instrument settings: pre-focusing at 1.5 kV for 1 minute, followed by focusing at 3.0 kV for 8 minutes. Each mAb sample was analyzed in triplicate and detected for 10 seconds using native fluorescence with an excitation wavelength of 280 nm and emission detection at 320–450 nm. Peak integration was performed using Compass for iCE Version 4.0.0 (ProteinSimple, San Jose, CA), and summary data graphs were generated using GraphPad Prism 10.0.0.

### Size variant analysis via capillary electrophoresis sodium dodecyl sulfate

rCE-SDS and nrCE-SDS were performed as an orthogonal method to assess size variants arising from photo-stress. Briefly, 25 µg of each protein was diluted in 50 µL of Maurice CE-SDS PLUS 1× Sample Buffer (Cat# 046–567, Bio-Techne), containing either 650 nM beta-mercaptoethanol (rCE-SDS) (Cat# M6250-500 ML, Sigma-Aldrich) or 11.5 mM iodoacetamide (nrCE-SDS), as specified by the manufacturer. An internal CE-SDS size standard (Cat# 046–144, Bio-Techne) was added to each sample according to the manufacturer’s instructions.

Samples were incubated at 70°C for 10 minutes before separation using a Maurice CE-SDS Turbo Cartridge (Cat# PS-MC02-TS, Bio-Techne). For rCE-SDS, sample loading was performed at 3.5 kV for 8 seconds, followed by separation at 4.2 kV for 7.5 minutes. For nrCE-SDS, sample loading was also conducted at 3.5 kV for 8 seconds, with separation at 4.2 kV for 10 minutes. Size variants were detected by absorbance at 220 nm. Low molecular weight fragments of adalimumab products detected in nrCE-SDS electropherograms were assigned based on the relative migration times of a Waters protein standard mix (BEH200 SEC, Catalog# 186006518, Waters) that contains a variety of proteins ranging from a molecular weight of 17,000 Da-660,000 Da (Thyroglobulin MW = 660,000 Da; IgG MW = 150,000 Da; BSA MW = 66,400 Da; Myoglobin MW = 17,000 Da). The relative migration of the HC, LC and ngHC peaks of a reduced IgG standard (Maurice CE-SDS IgG Standard, Catalog # 046–039, Protein Simple) were used to assign the major reduced peaks of adalimumab products in rCE-SDS electropherograms. Peak integration was performed using Compass for iCE Version 4.0.0 (ProteinSimple, San Jose, CA), and summary data graphs were generated using GraphPad Prism 10.0.0.

## Supplementary Material

Supplemental Figures 1-10.pdf

## Abbreviations

CCS: container closure system;
CCSD: Combined Chemical Shift Deviation;
CE-SDS: capillary electrophoresis sodium dodecyl sulfate;
CQA: critical quality attributes;
ECHOS: easy comparability of HOS;
Far-UV CD: Far-ultraviolet circular dichroism;
FDA: Food and Drug Administration;
FTIR: Fourier-transform infrared;
HMQC: heteronuclear multiple quantum coherence;
HMWS: high molecular weight species;
HOS: higher-order structure;
icIEF: imaged capillary isoelectric focusing;
LC-MS: liquid chromatography-mass spectrometry;
LMWS: low molecular weight species;
mAb: monoclonal antibody;
NMR: nuclear magnetic resonance;
1D: one-dimensional;
Ox-Met: oxidized methionine;
PCA: principal component analysis;
PFGSTE: pulsed-field gradient stimulated echo;
PLS: partial least squares;
PROFILE: protein fingerprint by line shape enhancement;
PS: polysorbate;
PTM: post-translational modification;
SEC: size exclusion chromatography;
2D: two-dimensional.
