# Supplementary material for: NMR-based structural integrity analysis of therapeutic monoclonal antibodies: a comparative study of Humira and its biosimilars
Source: MAbs. 2025 Sep 8;17(1):2551208. doi: 10.1080/19420862.2025.2551208 (PMC12427497; doi:10.1080/19420862.2025.2551208)
Supplement: Supplemental Figures 1-10.pdf [file KMAB_A_2551208_SM5077.pdf]

NMR-based structural integrity analysis of therapeutic monoclonal antibodies: A comparative study of Humira and its biosimilars

Donna Baldisseri[1,5], Shen Luo[2,5], Christelle Anne F. Ancajas[2], Uriel Ortega-Rodriguez[2], Christian Fischer[3], Guozhang Zou[2], Jianghong Gu[2], David Keire[2], Martial Piotto[4,6], Baolin Zhang[2,6]

[1]Bruker BioSpin Corp., Billerica, MA 01821, United States

[2]Office of Pharmaceutical Quality Research, Office of Pharmaceutical Quality, Center for Drug Evaluation and Research, Food and Drug Administration, Silver Spring, MD 20993, United States

[3]Bruker BioSpin Corp., 76275 Ettlingen, Germany

[4]Bruker BioSpin Corp., 67160 Wissembourg, France

[5]Equally contributed

[6] Correspondence:

Baolin Zhang, Ph.D., baolin.zhang@fda.hhs.gov, 240-402-6740

Martial Piotto, Ph.D., martial.piotto@bruker.com, (33)388-736-862

Supplemental Figure 1

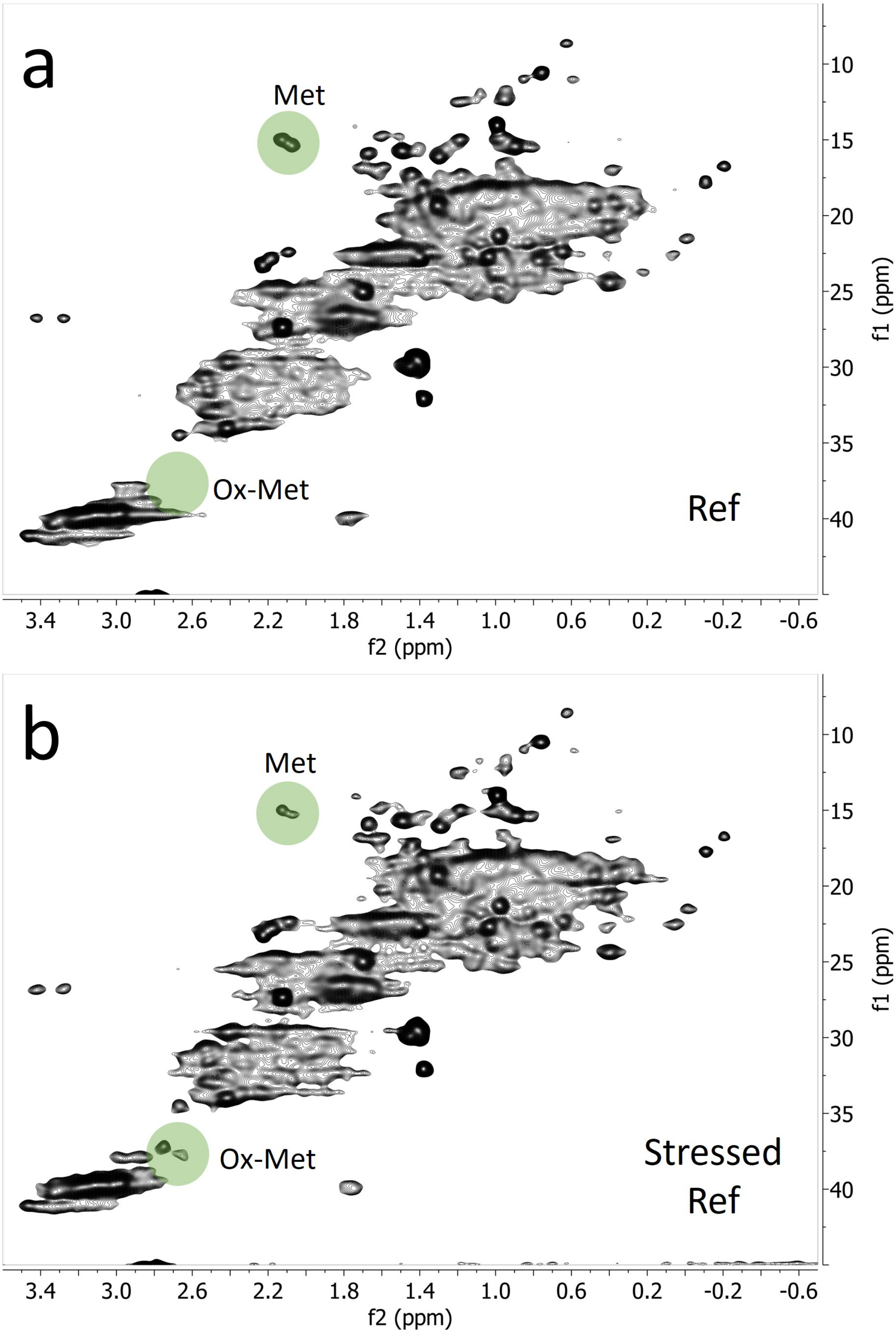

Supplemental Figure 1. 2D NMR spectra of the reference adalimumab under normal and photo-stressed conditions. Comparison of the 2D ^{1}H-^{13}C spectrum of the reference adalimumab under normal (a) and photo-stressed (b) conditions. The two spectral regions corresponding to Met and oxidized Met (Ox-Met) residues are highlighted.

Supplemental Figure 2

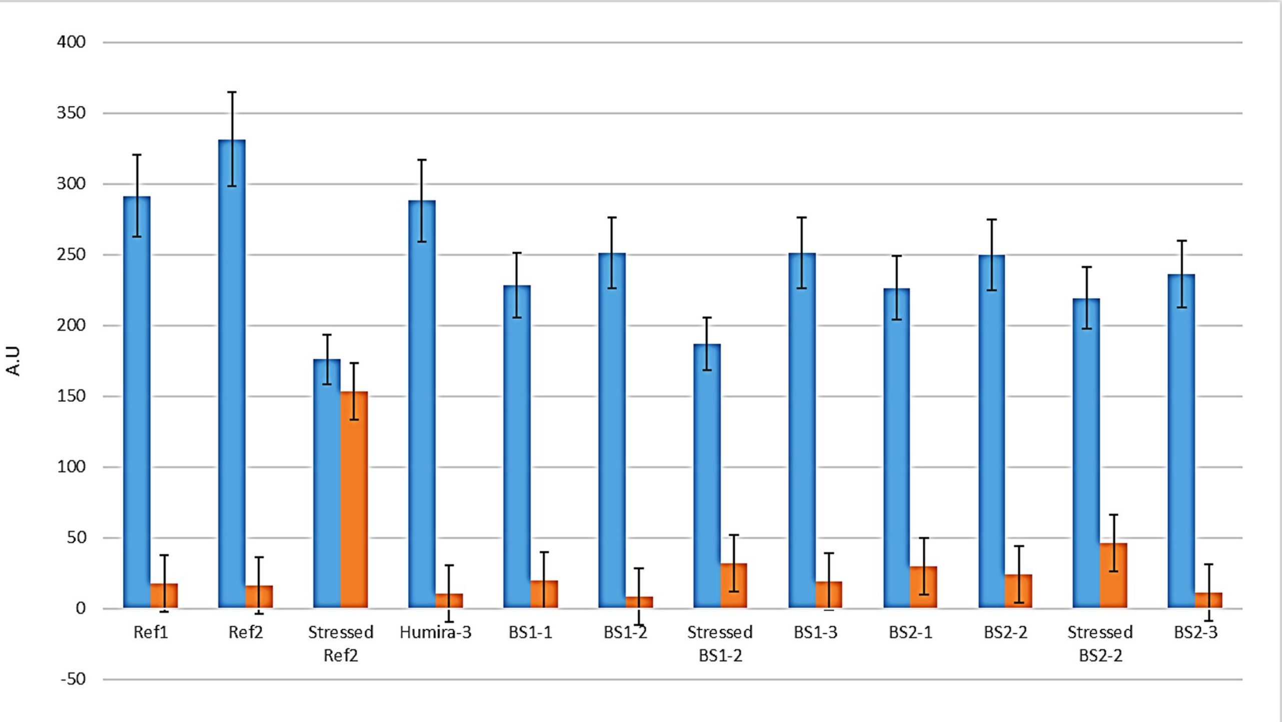

Supplemental Figure 2. Evolution of the quantity of Met (blue) and Ox-Met (orange) detected by 2D ^{1}H-^{13}C NMR in reference adalimumab, BS1 and BS2 for unstressed and photo-stressed samples. The visual inspection of the spectra shows that only Stressed Ref2, Stressed BS1-2 and Stressed BS2-2 samples contain Ox-Met. The small integral value measured for the Ox-Met region for the other samples is due to integration noise. The error bar corresponds to this integration noise.

Supplemental Figure 3

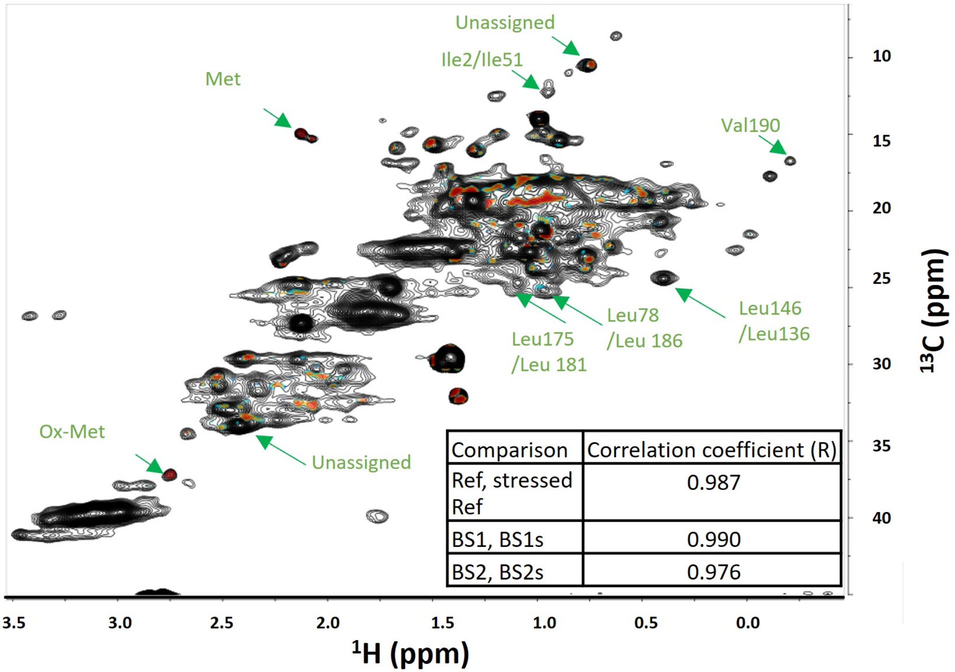

Comparison	Correlation coefficient (R)
Ref, stressed Ref	0.987
BS1, BS1s	0.990
BS2, BS2s	0.976

Supplemental Figure 3. ECHOS heat map for unstressed and stressed reference adalimumab. Inset table shows correlation coefficients calculated from ECHOS regression analysis of the comparisons indicated. The syntax Leu175/Leu181 indicates that there is an ambiguity in the assignment between Leu175 and Leu181. The same syntax applies to Ile2/Ile51, Leu78/Leu186 and Leu146/Leu136.

Supplemental Figure 4

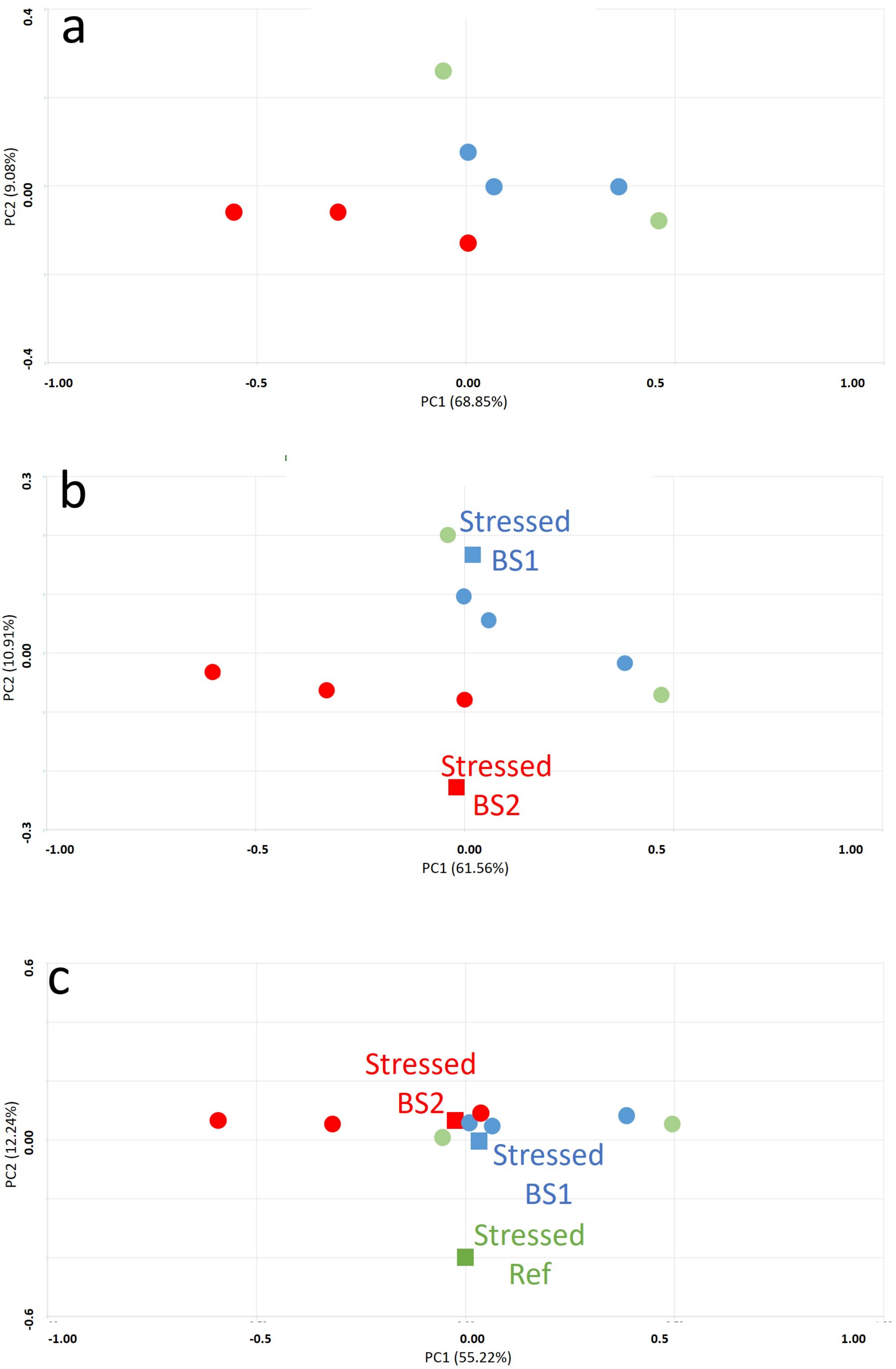

Supplemental Figure 4: PC1-PC2 scores plot of the PCA analysis of unstressed samples (a), unstressed and photo-stressed samples excluding (b) and including (c) the stressed reference adalimumab. The colored markers represent unstressed (circles) and stressed (squares) samples of reference adalimumab (green), BS1 (blue), and BS2 (red).

Supplemental Figure 5

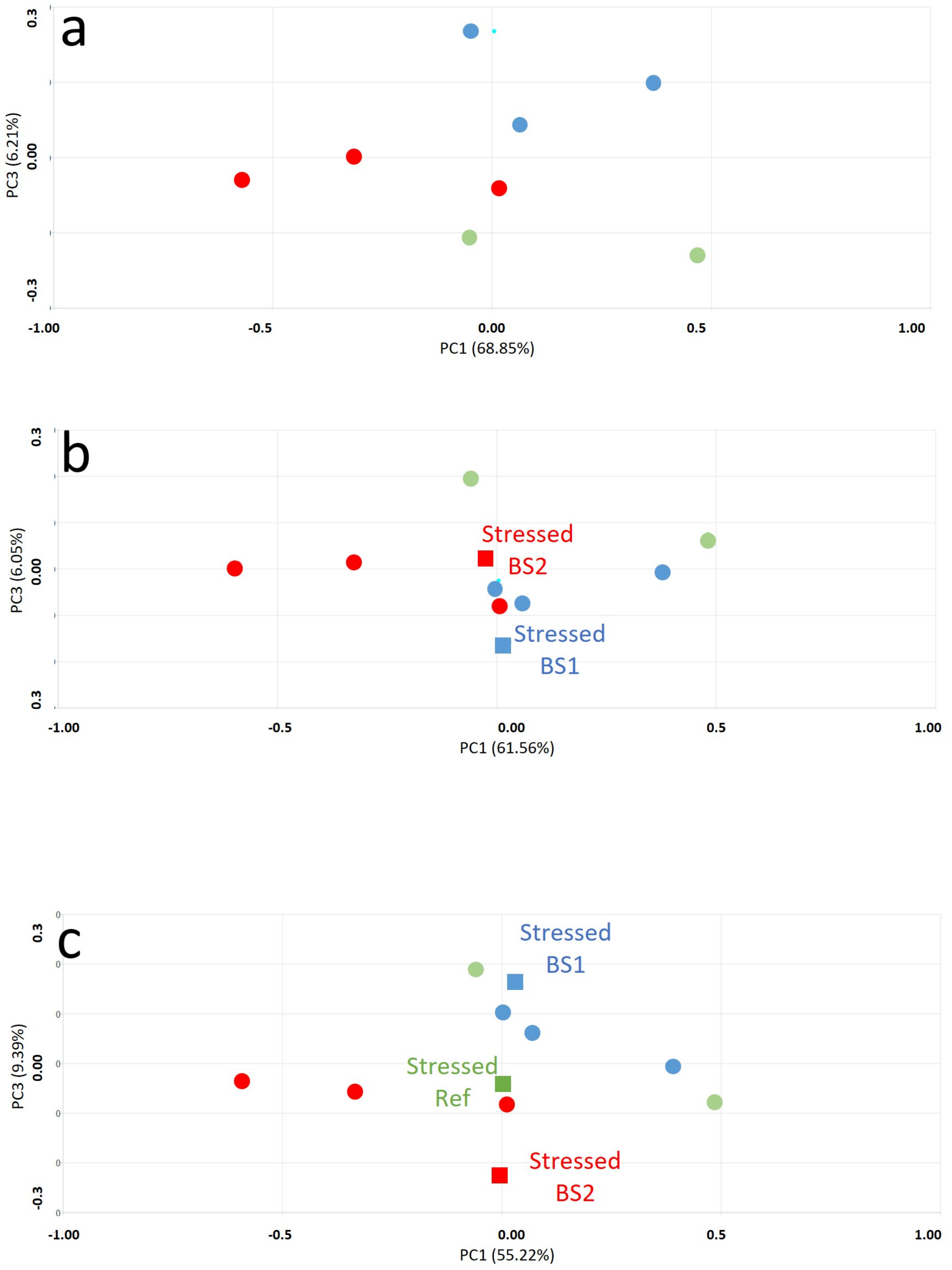

Supplemental Figure 5: PC1-PC3 scores plot of the PCA analysis of unstressed samples (a), unstressed and photo-stressed samples excluding (b) and including (c) the stressed reference adalimumab. The colored markers represent unstressed (circles) and stressed (squares) samples of reference adalimumab (green), BS1 (blue), and BS2 (red).

Supplemental Figure 6

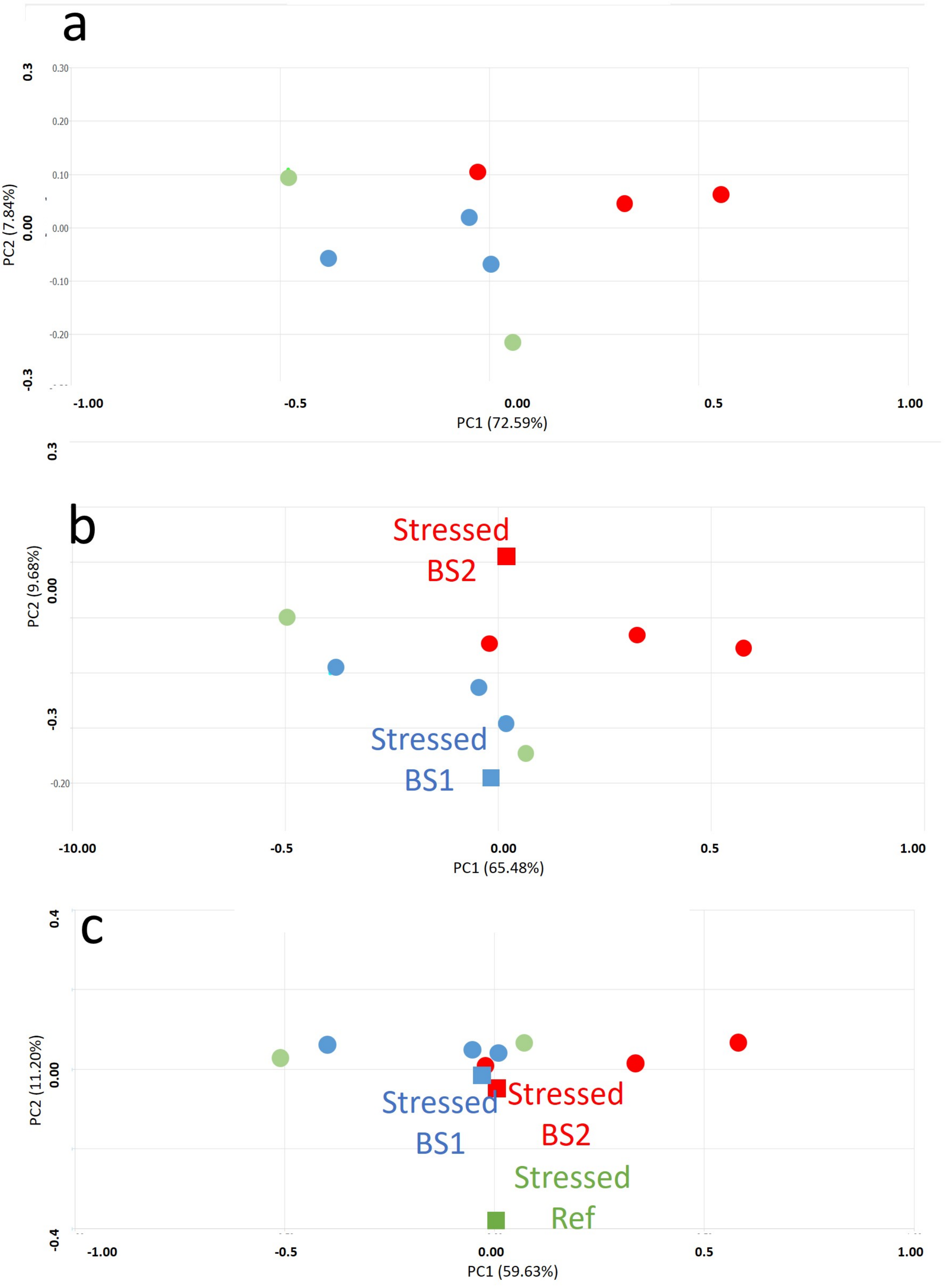

Supplemental Figure 6: PCA analysis taking into account only the methyl regions (without the methylene regions). PC1-PC2 scores plot of unstressed samples (a), unstressed and photo-stressed samples excluding (b) and including (c) the stressed reference adalimumab. The colored markers represent unstressed (circles) and stressed (squares) samples of reference adalimumab (green), BS1 (blue), and BS2 (red).

Supplemental Figure 7

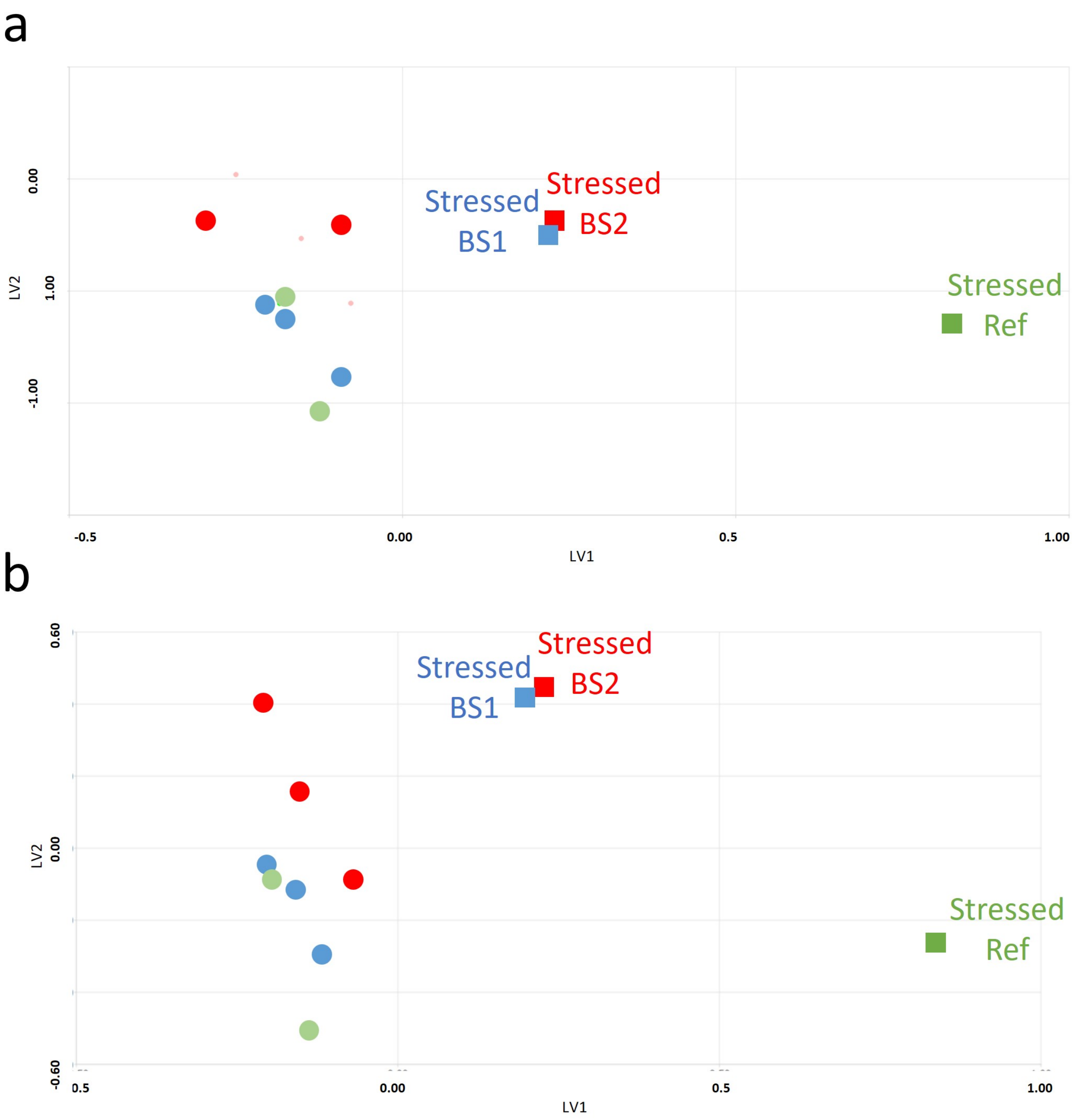

Supplemental Figure 7. PLS analysis of unstressed and photo-stressed samples with (a) and without (b) the regions corresponding to Met and Ox-Met signals. The colored markers represent unstressed (circles) and stressed (squares) samples of reference adalimumab (green), BS1 (blue), and BS2 (red).

Supplemental Figure 8

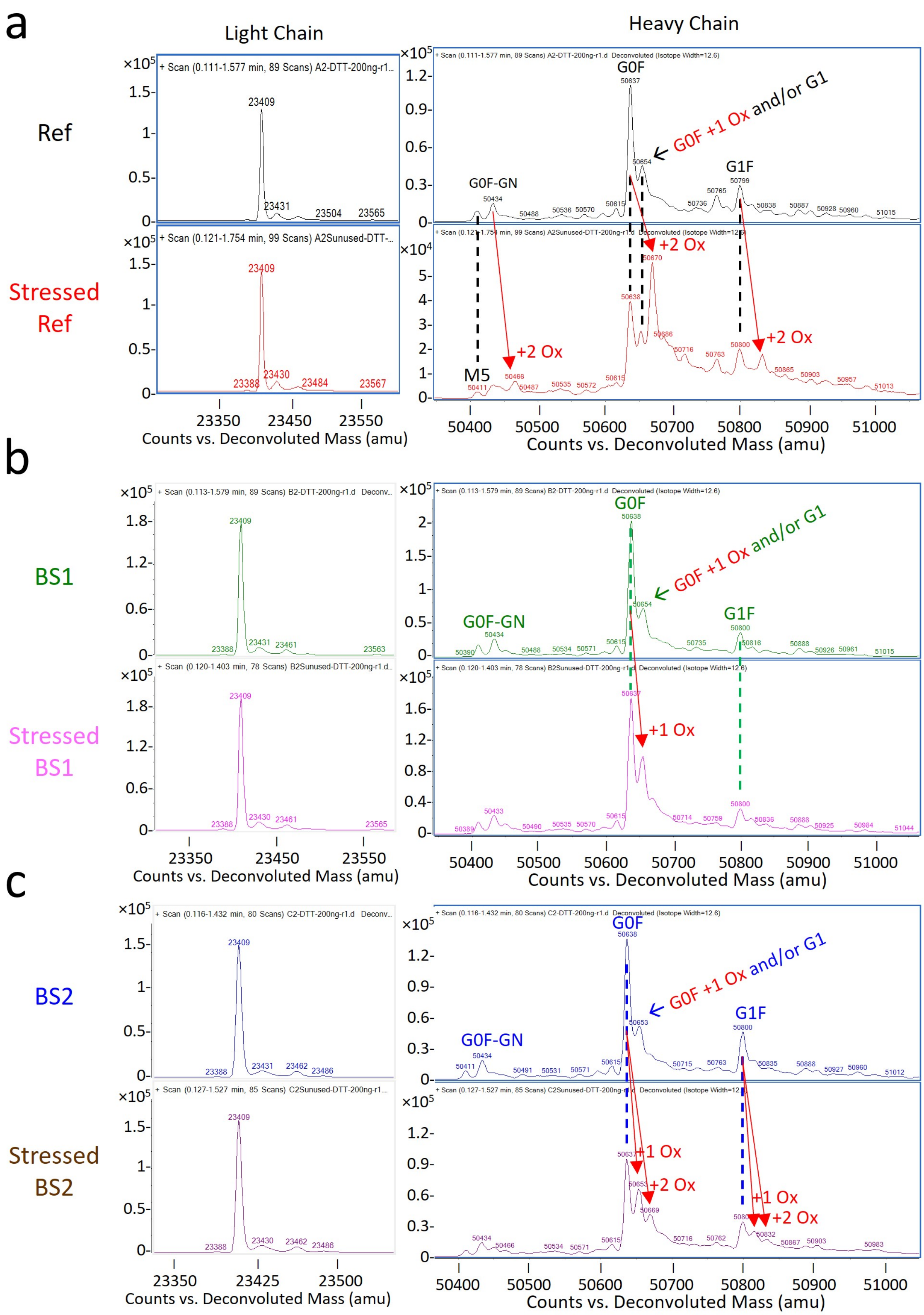

Supplemental Figure 8. Oxidation analysis by reduced LC-MS. The deconvoluted mass spectra of light and heavy chain for the indicated samples are shown in panels a to c. The major peaks are labelled by attached N-glycans. Mass peak with an increase of approximate 16 or 32 Da from a major peak is labelled as +1 or +2 oxygen (Ox), respectively, suggesting oxidation of one or two methionine residues. GN represents GlcNAc.

Supplemental Figure 9

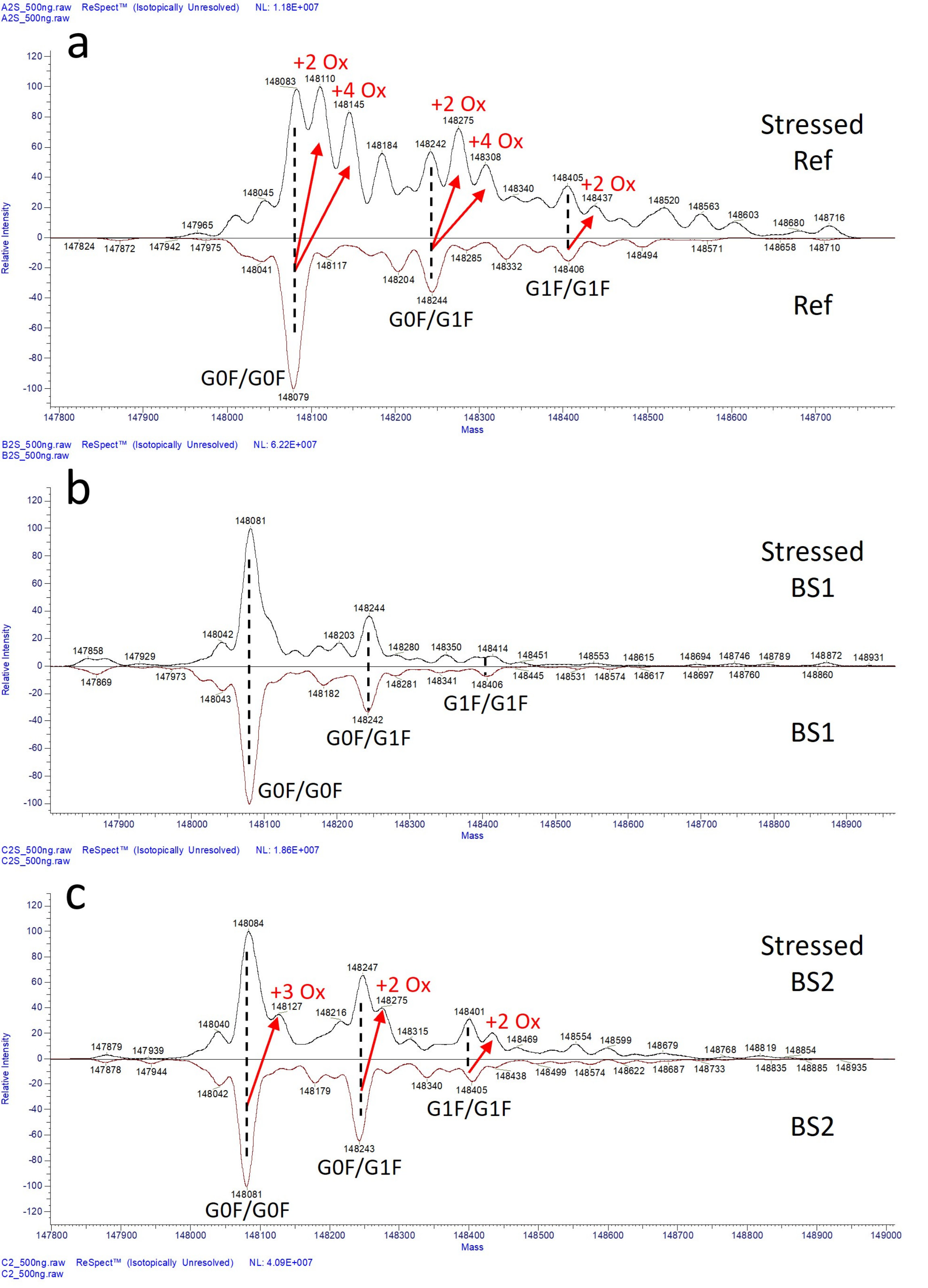

Supplemental Figure 9. Oxidation analysis by intact LC-MS. Shown are the deconvoluted mass spectra of the reference adalimumab (a), BS1 (b), and BS2 (c) under normal and photo-stressed conditions. Mass peak with an increase of approximate 32, 48 or 64 Da from a major peak is labelled as +2, +3 or +4 oxygen (Ox), respectively, suggesting oxidation of two, three or four methionine residues.

Supplemental Figure 10

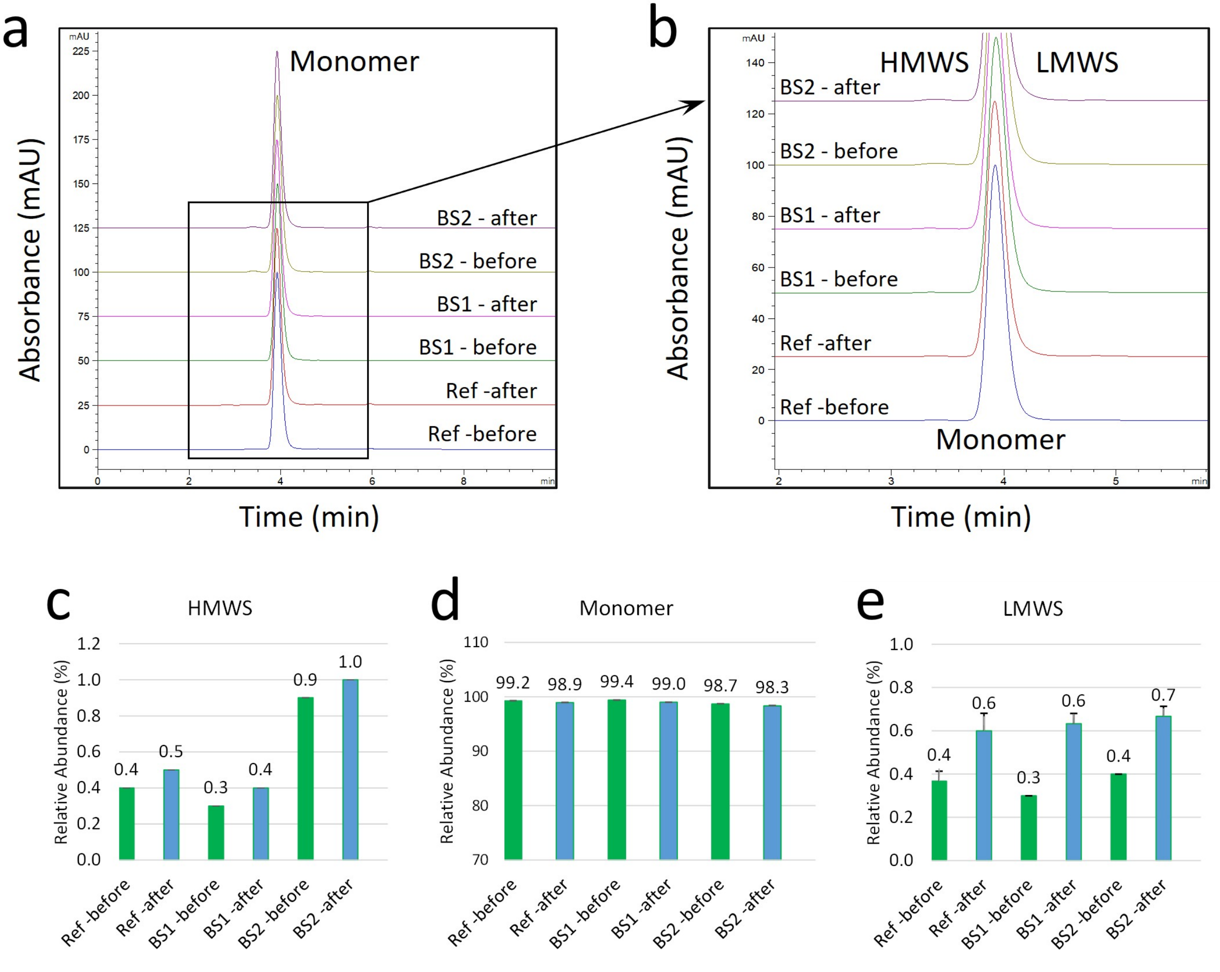

Supplemental Figure 10. Size variant analysis of the reference adalimumab and biosimilars by SEC. (a) SEC profiles showing separation of monomer, high molecular weight species (HMWS), and low molecular weight species (LMWS) in samples before and after NMR measurement at 45°C for 7 hours; boxed region enlarged in (b). (c–e) Relative abundances of HMWS, monomer, and LMWS, respectively, calculated from peak areas. Error bars represent standard deviation (n = 3).
